# Blood-based biomarkers in patients with non-small cell lung cancer treated with immune checkpoint blockade

**DOI:** 10.1186/s13046-024-02969-1

**Published:** 2024-03-16

**Authors:** Yo-Ting Tsai, Jeffrey Schlom, Renee N. Donahue

**Affiliations:** Center for Immuno-Oncology, Center for Cancer Research, National Cancer Institute, National Institutes of Health, Bethesda, MD USA

**Keywords:** Biomarker, Peripheral blood, Liquid biopsy, NSCLC, Immunotherapy

## Abstract

The paradigm of non-small cell lung cancer (NSCLC) treatment has been profoundly influenced by the development of immune checkpoint inhibitors (ICI), but the range of clinical responses observed among patients poses significant challenges. To date, analyses of tumor biopsies are the only parameter used to guide prognosis to ICI therapy. Tumor biopsies, however, are often difficult to obtain and tissue-based biomarkers are limited by intratumoral heterogeneity and temporal variability. In response, there has been a growing emphasis on the development of “liquid biopsy”‒ derived biomarkers, which offer a minimally invasive means to dynamically monitor the immune status of NSCLC patients either before and/or during the course of treatment. Here we review studies in which multiple blood-based biomarkers encompassing circulating soluble analytes, immune cell subsets, circulating tumor DNA, blood-based tumor mutational burden, and circulating tumor cells have shown promising associations with the clinical response of NSCLC patients to ICI therapy. These investigations have unveiled compelling correlations between the peripheral immune status of patients both before and during ICI therapy and patient outcomes, which include response rates, progression-free survival, and overall survival. There is need for rigorous validation and standardization of these blood-based assays for broader clinical application. Integration of multiple blood-based biomarkers into comprehensive panels or algorithms also has the potential to enhance predictive accuracy. Further research aimed at longitudinal monitoring of circulating biomarkers is also crucial to comprehend immune dynamics and resistance mechanisms and should be used alongside tissue-based methods that interrogate the tumor microenvironment to guide treatment decisions and may inform on the development of novel therapeutic strategies. The data reviewed here reinforce the opportunity to refine patient stratification, optimize treatments, and improve outcomes not only in NSCLC but also in the wider spectrum of solid tumors undergoing immunotherapy.

**Supplementary Information:**

The online version contains supplementary material available at 10.1186/s13046-024-02969-1.

## Introduction

### Current NSCLC therapies and the role of immunotherapy

Non-small cell lung cancer (NSCLC) accounts for approximately 80–85% of all lung cancer cases and remains the leading cause of cancer-related deaths [1]. A multifaceted approach is used for the treatment of NSCLC, with current approved treatment modalities including surgical resection, chemotherapy, radiation therapy, targeted therapies for patients with specific actionable genetic mutations, and immunotherapy with immune checkpoint inhibitors (ICI) [2, 3]. Programmed death-ligand 1 (PD-L1) is a membrane-bound protein, often found on tumor cells and normally expressed on multiple immune cells including T cells, B cells, dendritic cells and macrophages. Binding of tumor PD-L1 to its receptor, programmed death 1 (PD-1), a trans-membrane glycoprotein belonging to the B7 superfamily that is expressed on lymphocytes, dendritic cells, and activated monocytes, induces inhibitory signals to suppress the immune response, thus allowing tumor cells to escape from immunosurveillance [4–6]. Monoclonal antibodies blocking the interaction of PD-1 and PD-L1 activate an individual’s immune system to attack their cancer and have revolutionized the landscape of cancer treatment, showing clinical benefit in multiple cancers, including NSCLC. The U.S. Food and Drug Administration has approved several inhibitors of the PD-1/PD-L1 axis, including nivolumab, pembrolizumab, atezolizumab and durvalumab as monotherapy or in combination with platinum-based chemotherapy for a subset of patients with NSCLC. Treatment of NSCLC with ICI produces long-lasting survival and durable responses; however, responses are not seen in the majority of patients. Response rates of 19–45% are reported in patients treated with anti-PD-1 [7–10], and 18–36.4% with the combination of anti-CTLA-4 with anti-PD-1 [11–14].

### Current status of tissue-based biomarkers in NSCLC

Tumor-based biomarkers have been extensively investigated to guide the use of ICIs in NSCLC. Expression of PD-L1 in tumor biopsies, as assessed by immunohistochemistry, is an approved biomarker to guide treatment decisions and predict the likelihood of response of NSCLC patients to ICI. Generally, high baseline PD-L1 expression associates with superior outcomes following ICI monotherapy in patients who failed standard chemotherapy [10, 15]. However, some patients with low or negative tumor PD-L1 expression respond well to ICI therapy, possibly due to intratumoral heterogeneity, the site of the tumor biopsy, temporal fluctuations in PD-L1 after prior therapy, and/or other factors. In addition, a lack of standardization in methods and optimal cut-off values makes the use of tumor PD-L1 a controversial predictive marker of response to ICI.

Tumor mutational burden (TMB), defined as the total number of somatic mutations per megabase of the tumor genome, is another tissue-based biomarker that has been explored to predict clinical outcomes to ICI [16]. It is postulated that more neoantigens induce greater immune activation, with some data supporting the hypothesis that higher TMB correlates with improved responses. Hellmann et al. reported clinical benefit of nivolumab combined with ipilimumab in NSCLC patients with a high TMB irrespective of tumor PD-L1 expression [17, 18]. In line with this finding, a study of 2234 participants with advanced solid tumors including lung cancer found that among patients treated with pembrolizumab (*n* = 1772), patients with a high TMB (≥ 175 mutations/exome) had a greater objective response rate (ORR, 31.4%) compared to patients with low TMB values (9.5%). The association between TMB and improved clinical benefit was seen regardless of PD-L1 expression and was not driven by specific tumor types [19]. However, other studies using the same cut-point have since observed that TMB does not associate with clinical response in patients with NSCLC treated with the combination of pembrolizumab and chemotherapy [20, 21]. These results are inconsistent, and the clinical utility of TMB remains controversial as a predictor of response to ICI in NSCLC.

Other tumor-based biomarkers are also being explored to predict clinical outcomes in response to ICI, including evaluation of neoantigen load, the frequency of tumor infiltrating immune cells, and expression of immune regulatory mRNA signatures [22]. However, tissue-based biopsies are often difficult to obtain and, perhaps more importantly, only pre-treatment biopsies are often available, with many obtained years prior to patients receiving ICI (Table 1). It is also known that most solid tumors are heterogeneous, evolve phenotypically with time, and may be altered by prior therapeutic regimens. Acquiring adequate tumor samples in advanced cancers, including NSCLC, can be challenging, and tumors obtained from different sites have been shown to vary. Longitudinal monitoring of tumor biopsies is often not feasible, and therefore does not capture the plasticity of interactions between the tumor and immune system under selective pressure of ICI.

**Table 1:** Limitations with biomarker discovery in tumor vs. blood-based biopsies

Limitation	Tumor biopsy	Liquid/blood-based biopsy
Heterogeneity	Intratumoral and intertumoral heterogeneity	Systemic, potentially reducing the impact of tumor heterogeneity
Invasiveness	Invasive, may pose risks, accessibility issues	Less invasive, minimal risks
Sampling Bias	Limited sample size, not representative	Less prone to sampling bias
Temporal Variability	Dynamic changes not easily captured	Dynamic changes easily captured
Tissue Availability	Limited by biopsy location	Readily available
Technical Challenges	Technically challenging, degradation, contamination, quality issues	Less technically challenging with well-established protocols
Tissue Preservation	Crucial for biomarker integrity	Simple storage for future analysis
Ethical and Consent Issues	Obtaining informed consent can be complex	Fewer ethical and consent-related challenges
Cost and Resource Intensity	Expensive, requires specialized equipment	More cost-effective, requiring less specialized equipment
Data Integration and Interpretation Challenges	Complex analysis	More straightforward analysis

### Emerging role of blood-based biomarkers in NSCLC

**Fig. 1:**
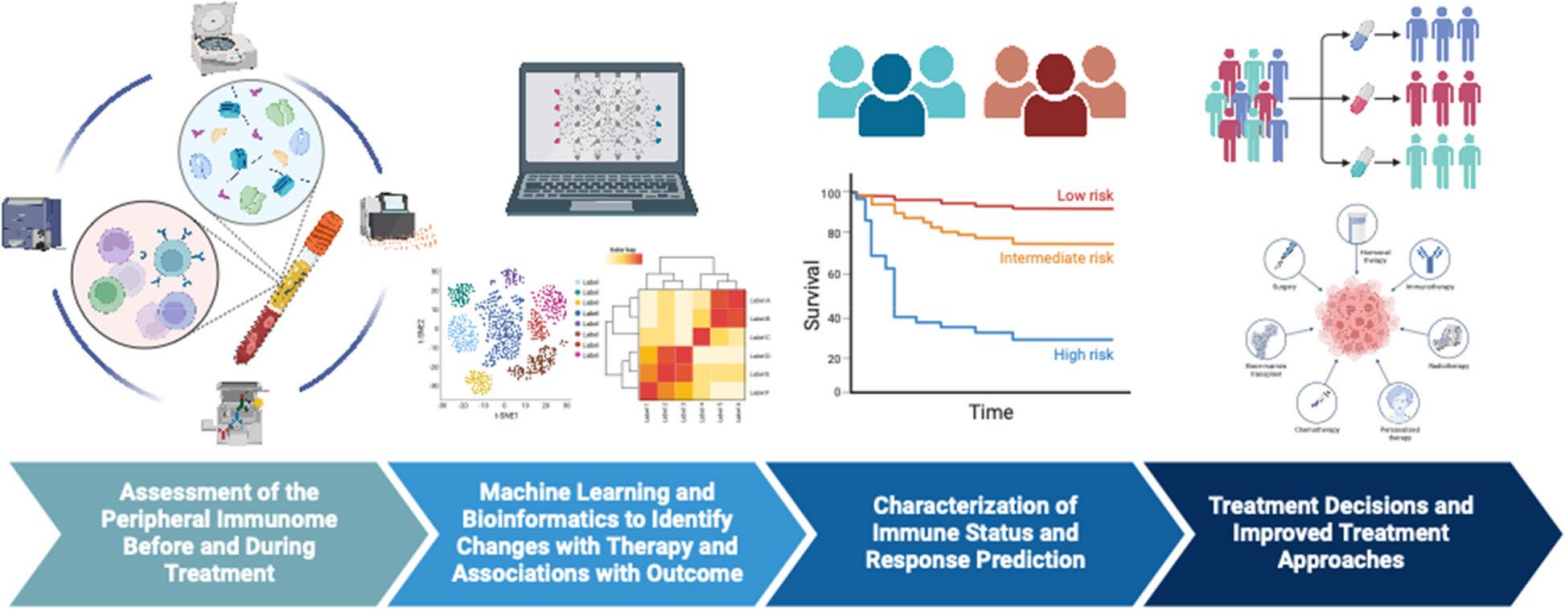
Clinical application of blood-based biomarkers in NSCLC. This figure showcases the potential clinical application of blood-based biomarkers in NSCLC patients treated with immunotherapy. Blood-based biomarkers may have a potential role in understanding the biology of a given agent in humans, patient selection, monitoring of treatment, and prediction of patient outcomes in response to therapy. (Created with BioRender.com)

In parallel with efforts to identify tumor-based biomarkers, an enormous effort has been made recently to develop blood-based biomarkers to predict patient response to ICI. Investigation of the status and activity of the systemic immune system is non-invasive, allows for relatively easy and dynamic profiling throughout treatment, and should be used to complement methods that directly interrogate the tumor microenvironment (Table 1). Blood-based biomarkers under evaluation include assays that interrogate (a) circulating levels of cytokines and other soluble factors, (b) exosomes, (c) phenotypes and frequencies of peripheral immune cell subsets, (d) levels of total circulating cell-free DNA (cfDNA) or circulating tumor DNA (ctDNA), (e) blood-based tumor mutational burden (bTMB), (f) microRNA (miRNA), which is involved in epigenetic regulation, and (g) circulating tumor cells (CTCs). These studies have revealed compelling associations between the immune status of patients both prior to immunotherapy and early during the course of therapy, and clinical responses such as overall response rates and the duration of progression-free and overall survival. This review focuses on recent advances in blood-based biomarkers that are potentially predictive of clinical response in NSCLC patients treated with ICI. None of these assays, however, are specific to NSCLC and could be considered more widely for application in immunotherapy studies of all solid tumors. Standardization of these assays along with integration of multiple biomarkers and computational bioinformatic studies will be needed to facilitate the clinical application of blood-based biomarkers in NSCLC treated with ICI (Fig. 1). This is an emerging field, and this review illustrates the potential of diverse blood-based biomarkers to refine patient stratification to predict therapeutic response and improve long-term outcomes of NSCLC patients treated with ICI.

## Circulating levels of cytokines and soluble factors 

Numerous studies have evaluated the value of measuring various proteins circulating in the serum or plasma of patients with NSCLC treated with ICI. As detailed below, some circulating analytes may have prognostic value based on levels prior to ICI, while for others, early changes during therapy associate with patient benefit.

### High baseline levels of IL-6 associate with poor clinical outcome

**Fig. 2:**
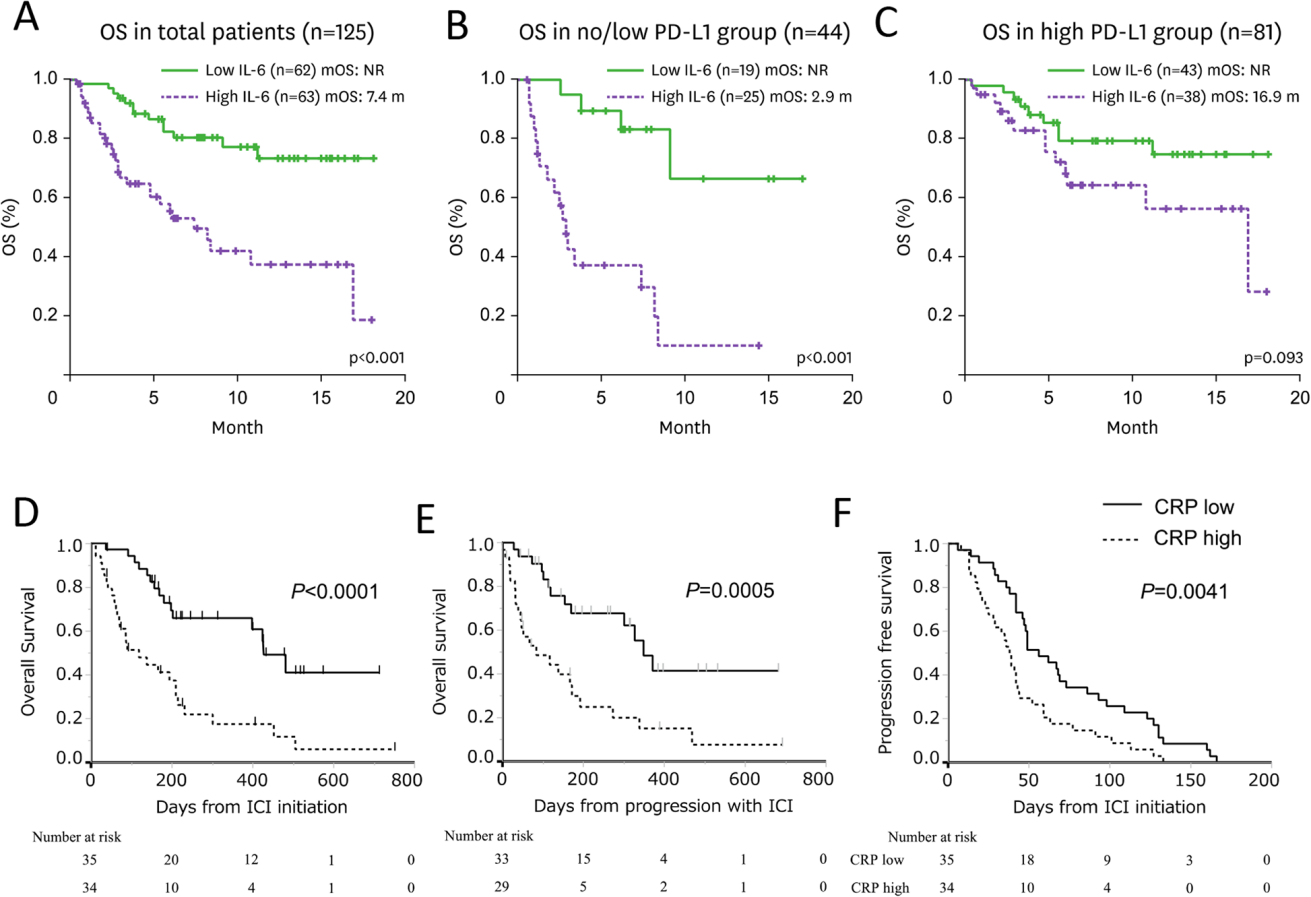
Survival analysis based on pre-therapy levels of serum IL-6 and CRP in NSCLC patients treated with PD-1/PD-L1 inhibitors. Overall survival (OS) according to baseline serum IL-6 levels (high vs. low) in NSCLC patients treated with anti-PD-1/anti-PD-L1 (**A**-**C**). OS in all patients (**A**), in patients with no/low PD-L1 tumor expression (**B**), and in patients with high PD-L1 tumor expression (**C**). Survival outcomes in patients with NSCLC without durable clinical benefit following anti-PD-(L)1, according to baseline CRP levels (**D-F**). OS from the start of immune checkpoint inhibitors (**D**), OS from time of progression with immune checkpoint inhibitors (**E**), and PFS from the start of immune checkpoint inhibitors (**F**). **A**-**C** modified from Kang, ref. [27]; copyright © 2020. The Korean Association of Immunologists. **D**-**F** modified from Harutani, ref. [38]; copyright © 2022, Springer Nature

Interleukin 6 (IL-6), an inflammatory cytokine, plays an important role in the stimulation of acute phase responses; however, dysregulation of IL-6 is associated with cancer progression and resistance [23, 24]. Five different studies involving 342 patients have shown that high levels of IL-6 at baseline (prior to ICI therapy) correlated with shorter progression-free survival (PFS) and in many cases lower response rates and inferior overall survival (OS) for patients receiving ICI (Table 2). Specifically, a study in stage III/IV NSCLC patients (*n* = 103) found that high baseline serum IL-6 (> 13.8 pg/ml) associated with worse PFS (*p* = 0.007) and OS (*p* = 0.003) following anti-PD-L1 as monotherapy or in combination with chemotherapy [25]. In another study of 29 stage IV NSCLC patients receiving anti-PD-1, patients with high baseline serum IL-6 (≥ 11.6 pg/ml) responded poorly (*p* < 0.001) and had a shorter PFS (5.14 vs. 38.57 weeks, *p* < 0.001) compared with patients with low IL-6 [26]. Furthermore, Kang and colleagues, in a study of 125 stage III/IV NSCLC patients treated with anti-PD-(L)1, found that low pre-therapy levels of serum IL-6 (< 13.1 pg/ml) associated with improved OS (*p* < 0.001) (Fig. 2A), with similar findings in subgroup analyses of patients with no/low tumor PD-L1 (Fig. 2B), or high tumor PD-L1 expression (Fig. 2C) [27]. In this study, a higher ORR (33.9% vs. 11.1%, *p* = 0.003) and disease control rate (DCR; 80.6% vs. 34.9%, *p* < 0.001) were also reported in patients with low IL-6 before treatment. In a recent study by Lambert and colleagues, low pre-treatment serum IL-6 associated with prolonged PFS (*p* = 0.011) in a univariate analysis of the anti-PD-1 inhibitor budigalimab in patients with advanced NSCLC (*n* = 40) or head and neck squamous cell carcinoma (HNSCC; *n* = 41) [28]. In another study using similar plasma cutoffs, a low baseline concentration of plasma IL-6 (< 11.150 pg/ml) associated with longer PFS (*p* = 0.0142) following anti-PD-1 therapies in stage III-IV patients with NSCLC [29].

**Table 2:** Association between IL-6, IL-8 and sPD-L1 at baseline and clinical outcome after ICI

Biomarker		NSCLC stage (n)	Treatment	Direction at baseline	Association with clinical outcome			Ref
					**Response**	**PFS**	**OS**	
**Cytokine**	**IL-6**	III-IV (*n* = 103)	αPD-1, ﻿αPD-L1, αPD-1/chemo, ﻿αPD-L1/ chemo	↑		↓ (*p* = 0.007)	↓ (*p* = 0.003)	[25]
		IV (*n* = 29)	αPD-1	↑	↓ (*p* < 0.001)	↓ (*p* < 0.001)		[26]
		III-IV (*n* = 125)	αPD-1, αPD-L1	↑	↓ (*p* = 0.003)	↓ (*p* < 0.001)	↓ (*p* < 0.001)	[27]
		Advanced NSCLC (*n* = 40), HNSCC (*n* = 41)	αPD-1	↑	↓ (*p* = 0.035)	↓ (*p* = 0.011)		[28]
		III-IV (*n* = 45)	αPD-1	↑	↓ (*p* = 0.018)	↓ (*p* = 0.0142)		[29]
	**IL-8**	IIIB/IV squamous (*n* = 108)	αPD-1	↑			↓ (*p* = 0.0051)	[30]
		IIIB/IV nonsquamous (*n* = 255)		↑			↓ (*p* < 0.0001)	
		IV (*n* = 29)	αPD-1	↑	↓ (*p* = 0.006)	↓ (*p* = 0.030)		[26]
		IIIB-IV (*n* = 143)	αPD-1, αPD-L1, αPD-1/chemo	↑	↓ (*p* < 0.05)			[31]
**Soluble Factor**	**sPD-L1**	IV solid tumor (*n* = 128, 50 with NSCLC)	ICI, ICI/other	↑	↓ (*p* = 0.013)	↓ (*p* = 0.023)	↓ (*p* = 0.005)	[32]
		IV or recurrent (*n* = 39)	αPD-1	↑	↓ (*p* = 0.0069)	↓ (*p* = 0.032)	↓ (*p* = 0.040)	[33]
		Metastatic (*n* = 51)	αPD-1	Positive		↓ (*p* = 0.004)	↓ (*p* = 0.013)	[34]
		Lung cancer (*n* = 485, from 6 studies)	ICI	↑	ns	↓ (*p* < 0.001)	↓ (*p* < 0.001)	[35]
		I-II (*n* = 3), III-IV (*n* = 40)	αPD-1	↑		↓ (*p* = 0.018)	↓ (*p* = 0.096)	[36]

*ICI* Immune checkpoint inhibitors, *OS* Overall survival, *ns* Not significant, *NSCLC* Non-small cell lung cancer, *PFS* Progression-free survival

C-reactive protein (CRP) is a non-specific acute phase protein that is induced by IL-6, and is a sensitive systemic marker of inflammation, infection, and tissue ﻿damage [37]. A study by Harutani and colleagues designed to explore the mechanism of action of prolonged OS in patients without initial apparent durable disease suppression after receiving ICI evaluated pre-therapy levels of multiple serum proteins in advanced NSCLC patients [38]. In that study, of 106 patients enrolled, 69 progressed or died within six months of ICI initiation and were classified as having non-durable clinical benefit. The authors found that before initiation of anti-PD-(L)1, the duration of OS (Fig. 2D), OS after progressive disease (PD; OS-PD, Fig. 2E), and PFS (Fig. 2F) was longer in the group of patients with CRP < 1.44 mg/dL. Collectively, these studies demonstrate that elevated circulating levels of IL-6, or CRP that is induced by IL-6, prior to therapy is associated with poor prognosis in NSCLC patients receiving ICI.

### Increases in IL-6 associate with poor clinical outcome

Three studies involving 293 patients reported that elevated levels or increases in IL-6 after ICI associate with poor clinical response (Table 3). Specifically, Harel et al., in 143 stage IIIB-IV NSCLC patients treated with anti-PD-(L)1 alone or in combination with chemotherapy, found a greater increase in IL-6 in non-responders than responders (*p* < 0.001) [31]. Similarly, Keegan and colleagues, in 47 metastatic NSCLC patients receiving anti-PD-(L)1 reported that patients with a > 40% increase or stable levels of IL-6 had reduced PFS compared to patients with decreases in IL-6 (median PFS: 4 vs. 11 months, *p* = 0.03) [39]. In that study, changes in IL-6 also associated with best overall response (BOR), with patients developing progressive disease (PD) having greater increases in IL-6 than patients with stable disease (SD) or partial response (PR) (*p* = 0.01), with the extent of change in IL-6 also correlating with the change in CRP. In the third study, although the post-treatment point was not specified, Shi et al. found that an increase in serum IL-6 (> 40% vs. baseline) after anti-PD-L1 alone or in combination with chemotherapy associated with inferior OS (*p* < 0.001) in stage III-IV NSCLC patients (*n* = 103) [25]. Overall, these studies demonstrate that increases in IL-6 in NSCLC patients following ICI associate with poor clinical response.

**Table 3:** Association between IL-6 and IL-8 after ICI therapy and clinical outcome

Biomarker		NSCLC stage (n)	Treatment	Direction after treatment	Association with clinical outcome			Ref
					**Response**	**PFS**	**OS**	
**Cytokine**	**IL-6**	III-IV (*n* = 103)	αPD-1, αPD-L1, αPD-1/chemo, αPD-L1/chemo	↑			↓ (*p* < 0.001)	[25]
		Metastatic (*n* = 47)	αPD-1, αPD-L1	↑	↓ (*p* = 0.01)	↓ (*p* = 0.03)		[39]
		IIIB-IV (*n* = 143)	αPD-1, αPD-L1, αPD-1/chemo	↑	↓ (*p* < 0.001)			[31]
	**IL-8**	Metastatic (*n* = 19)	αPD-1	↑	↓ (*p* < 0.0001)		↓ (*p* = 0.004)	[40]
		II-IV (*n* = 27)	αPD-1	↑		ns	↓ (*p* = 0.025)	[41]
		III-IV (*n* = 44)	αPD-1/radiotherapy	↑	↓ (*p* < 0.05)		↓ (*p* = 0.0058)	[42]
		IIIB-IV (*n* = 143)	αPD-1, αPD-L1, αPD-1/chemo	↑	↓ (*p* < 0.05)		↓ (*p* = 0.0146)	[31]
				↑ IL-8 and CXCL10 (model)			↓ (*p* = 0.087)	
		II-IV lung cancer (*n* = 67, with NSCLC, *n* = 57)	αPD-1/chemo	↑ CXCL10:IL-8		↑ (*p* = 0.0005)	↑ (*p* = 0.0006)	[43]

*ICI* Immune checkpoint inhibitors, *OS* Overall survival, *ns* Not significant, *NSCLC* Non-small cell lung cancer, *PFS* Progression-free survival

### High baseline levels of IL-8 associate with poor clinical outcome

**Fig. 3:**
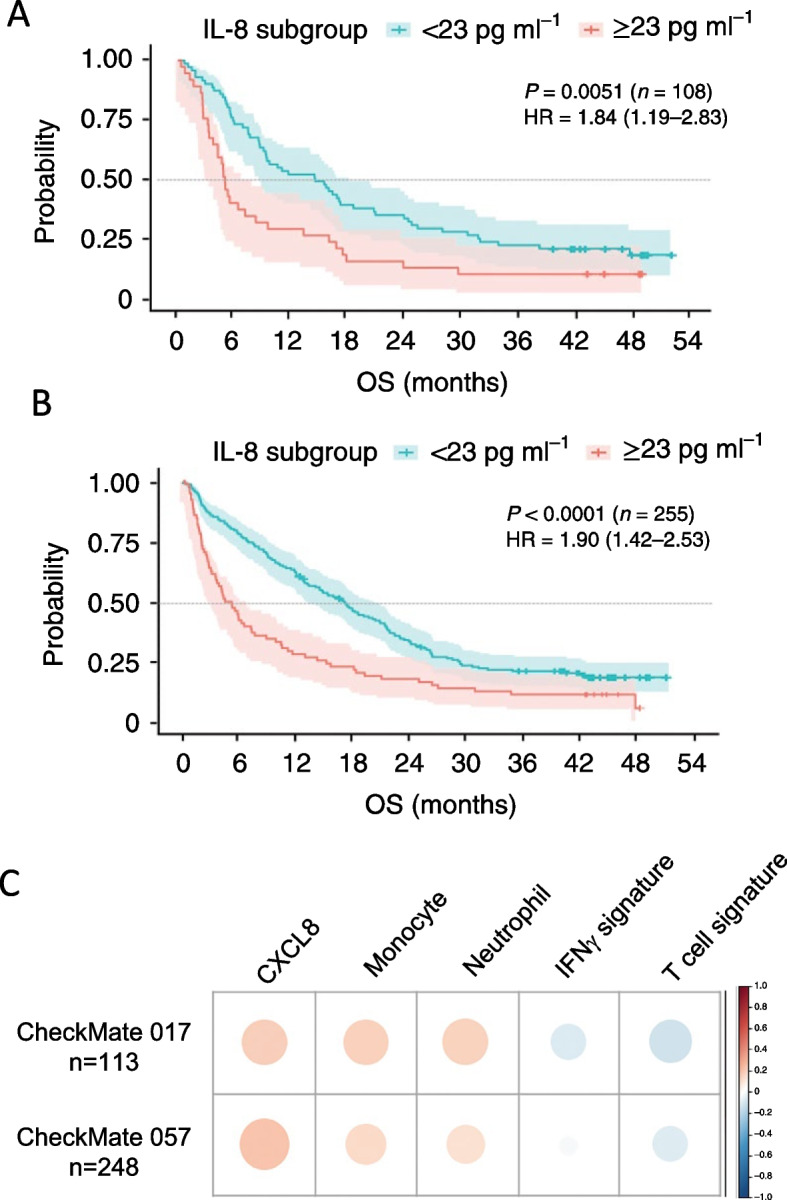
Association between IL-8 levels and survival and monocytes and neutrophils in NSCLC patients treated with nivolumab. Overall survival analysis of baseline serum IL-8 levels in CheckMate 017 (**A**) and CheckMate 057 (**B**) trials. **C** Association between serum IL-8 levels and tumor mRNA expression of CXCL8, peripheral monocyte counts, blood neutrophil counts, IFNγ-related gene signature and the T-cell-related mRNA signature in patients with NSCLC (CheckMate 017 and CheckMate 057). The color intensity of the circles indicates the Spearman’s correlation coefficient of the association. Darker red, larger dots represent a greater correlation between serum IL-8 level and the indicated factor; darker blue, larger dots represent a greater negative correlation between serum IL-8 level and the indicated factor. Modified from Schalper, ref. [30]; copyright © 2020, Springer Nature

Interleukin 8 (IL-8 or CXCL8), a chemokine secreted by myeloid, endothelial, epithelial, and tumor cells, attracts neutrophils to areas of inflammation. Its role as a poor prognostic indicator has been established in multiple cancers, with agents inhibiting the IL-8-CXCR1/CXCR2 axis under development for the treatment of cancer [44]. Multiple studies (*n* = 3) involving 535 patients have shown that high circulating baseline IL-8 prior to therapy associates with poor clinical response after ICI (Table 2). A large study in 1344 advanced cancer patients treated with nivolumab and/or ipilimumab, everolimus, or docetaxel, which included 363 NSCLC patients (squamous NSCLC, *n* = 108, from CheckMate 017; nonsquamous NSCLC, *n* = 255, from CheckMate 057), demonstrated that elevated serum IL-8 at baseline associated with poor outcome across treatment and cancer types [30]. High IL-8 at baseline (> 23 pg/ml) was associated with shorter OS in squamous NSCLC patients treated with nivolumab (Fig. 3A, CheckMate 017, *p* = 0.0051) and non-squamous NSCLC patients treated with nivolumab (Fig. 3B, CheckMate 057, *p* < 0.0001). A positive association between circulating IL-8 levels and tumor mRNA expression of CXCL8, and an inverse correlation with tumor IFNγ and T cell signatures were also reported (Fig. 3C), demonstrating that circulating levels of IL-8 are reflective of changes in the tumor microenvironment. In another study of stage IV NSCLC patients (*n* = 29), Kauffmann-Guerrero et al. found that individuals with higher baseline serum IL-8 (≥ 19.67 pg/ml) had a lower response rate (*p* = 0.006) and reduced PFS (*p* = 0.030, median PFS 4.0 weeks vs. 19.71 weeks) following anti-PD-1 than patients with IL-8 below this threshold [26]. Finally, in a study of 143 stage IIIB/IV NSCLC patients treated with anti-PD-(L)1 alone or in combination with chemotherapy, higher IL-8 levels were detected at baseline in non-responders than responders (*p* < 0.05) [31]. These studies consistently demonstrate that elevated levels of IL-8 prior to ICI in NSCLC patients are associated with poor prognosis.

### Increases in IL-8 after ICI associate with poor clinical outcome

Four different studies involving 233 lung cancer patients have shown that high levels or increases in IL-8 after ICI therapy associate with poor clinical response, which includes OS, and in many cases overall response rate (Table 3). In a study of 19 metastatic patients receiving anti-PD-1, serum IL-8 levels were decreased from baseline at the time of best response (20.8 pg/ml vs. 6.5 pg/ml, *p* = 0.005) in responders and increased at the time of disease progression (12 pg/ml vs. 51 pg/ml, *p* = 0.016) in non-responders [40]. The authors identified that a > 9.2% change 2–3 weeks after anti-PD-1 therapy predicted response with 85.7% sensitivity and 100% specificity. In addition, patients with ≥ 9.2% increase in IL-8 had a shorter OS than patients with either a decrease or a < 9.2% increase in this measure (median OS: 8 months vs. not reached at 21 months, *p* = 0.004). Agullo-Ortuno et al. also observed increased levels of circulating plasma IL-8 at time of disease progression in patients who initially responded to nivolumab but then progressed [41]. Here, at 2 months post-treatment, superior OS was seen in patients with a greater decrease in IL-8 compared with patients with an increase in IL-8 (median OS not reached at 20 months vs. 9.84 months, *p* = 0.025). In another study by Kang et al., in 44 patients with stage III-IV NSCLC, a decrease in plasma IL-8 three months following anti-PD-1 combined with hypo-fractionated radiotherapy was reported in patients with disease remission or stable disease, while patients with PD had increases in IL-8; lower levels of IL-8 after treatment were also associated with prolonged OS (*p* = 0.0058) [42]. These studies collectively demonstrate that increases in IL-8 after ICI associate inversely with patient response.

Two additional studies involving 200 NSCLC patients have shown that changes in the level of IL-8 along with the chemokine CXCL10 can serve as a prognostic indicator of response to ICI (Table 3). Harel et al., using proteomic analyses of ~ 800 pre- and on-treatment plasma proteins and machine learning algorithms, developed a predictive signature that showed a trend of reduced OS in stage IIIB/IV NSCLC patients (*n* = 143) treated with anti-PD-(L)1 monotherapy or chemoimmunotherapy based on the levels 2–6 weeks post-treatment of IL-8 and the chemokine CXCL10, along with two clinical parameters (age and sex) (Table 3) [31]. Another study of patients with stage II to IV lung cancer (*n* = 67, the majority with NSCLC) evaluated the association between changes in the ratio of plasma CXCL10 to IL-8 after treatment with nivolumab or pembrolizumab combined with chemotherapy [43]. Here, an increase in the ratio of plasma CXCL10/IL-8 after 10–12 weeks of therapy was associated with improved PFS (*p* = 0.00054) and OS (*p* = 0.00064).

### High baseline levels of sPD-L1 and increases after ICI associate with poor clinical outcome

The membrane-bound PD-1 and its ligand PD-L1 are well known to regulate host immune activity and function. Their soluble forms (sPD-1 or sPD-L1), produced by shedding or alternative splicing, retain their biological activity, and thus have the potential to regulate host immunity [45, 46]. Several studies have evaluated whether soluble PD-L1 levels associate with tumor expression of PD-L1 and found no correlation [32, 36, 47]; however, levels of these analytes have been detected in peripheral blood of cancer patients and shown prognostic significance across multiple tumor types [45, 48]. Pre-treatment levels of sPD-L1 in NSCLC patients have been assessed by multiple investigators as a predictive biomarker for ICI therapy, with elevated levels reported in five studies representing 668 patients demonstrating a negative association with PFS and OS [32–36] (Table 2). As an example, Tiako Meyo et al. found that prior to nivolumab treatment, 29.4% and 52.9% of patients were positive for sPD-L1 and sPD-1, respectively, and a composite biomarker consisting of sPD-L1 and sPD-1 positivity at baseline associated with both shorter PFS (*p* = 0.0002) and OS (*p* = 0.003) [34]. Furthermore, a recent meta-analysis of 1188 lung cancer patients reported that the optimal cut-off for sPD-L1 to discriminate responders from non-responders ranged from 27.22 pg/ml to 7.32 ng/ml [35]. In that analysis, high baseline sPD-L1 associated with both inferior PFS (*p* < 0.001) and OS (*p* < 0.001) in NSCLC patients treated with ICIs. In patients with advanced NSCLC (*n* = 43), Constantini et al. similarly found that patients with low sPD-L1 levels (< 33.97 pg/ml) had a longer PFS (*p* = 0.018) and a trend of longer OS (*p* = 0.096) following treatment with nivolumab than patients with high levels [36]. These studies collectively demonstrate that in NSCLC patients high baseline levels of sPD-L1 associate poorly with clinical response to ICI.

Three studies have also reported on the association between changes in levels of sPD-L1 after ICI in NSCLC patients and clinical response with mixed findings [36, 47, 49] (Supplemental Table 1). Additional circulating analytes such as sPD-1 [34, 50], interferon-gamma [26, 51–54], granzyme B [36, 55], sCD25 [56], lactate dehydrogenase [41, 57], L-Arginine [58], and indoleamine 2,3 dioxygenase [41] have been evaluated for association with outcome of NSCLC patients before and/or after ICI with some conflicting results (Supplemental Tables 1 and 2). Further studies in additional cohorts of NSCLC patients are needed to confirm their relevance.

## Serum proteomic tests at baseline associate with response to ICI

Several serum proteomic tests based on pre-therapy levels of soluble analytes have been developed, using machine learning algorithms to stratify patients with NSCLC treated with ICI to predict clinical outcomes [59–61] (Supplemental Table 2). Taguchi et al. developed a test based on eight mass spectral features called the Host Immune Classifier (HIC), in which patients with NSCLC were classified as HIC-hot (HIC-H), or HIC-cold (HIC-C) to represent patient immune status and predict response to epidermal growth factor receptor tyrosine kinase inhibitors, a test that is now commercially marketed as the VeriStrat test [62]. Rich and colleagues applied this test in a prospective study of 877 advanced NSCLC patients treated with first-line therapies, including ICI (*n* = 284) [59]. Here, patients classified as HIC-H had longer OS than HIC-C patients in cohorts receiving ICI monotherapy (*p* < 0.0001) or ICI combined with chemotherapy (*p* < 0.001). Of note, these findings were shown to be independent of PD-L1 tumor expression.

Another serum proteomic test, developed by Muller et al., consisting of 274 mass spectral features and called the Primary Immune Response (PIR) test, was generated in 116 advanced NSCLC patients treated with nivolumab [60]. Here, the PIR test stratified patients into three groups: sensitive, intermediate, and resistant to therapy. PFS for the treatment resistant group was shorter than for the other groups (1.4, 4.3, and 9.1 months for the resistant, intermediate, and sensitive groups, respectively, *p* < 0.001 for resistant vs. sensitive groups). This test also stratified patients by OS (4.3, 10.4, and 17.3 months reported for the resistant, intermediate, and sensitive groups, respectively; *p* < 0.001 for resistant vs. sensitive). The PIR test was further validated by Muller and colleagues in two additional cohorts (*n* = 98 and *n* = 75) of NSCLC patients treated with anti-PD-1; however, this test did not associate with PFS or OS in a historical cohort of second-line NSCLC patients treated with docetaxel. These proteomic tests discriminated patients with good versus poor clinical outcomes prior to immunotherapy; however, neither of these studies revealed the specific analytes incorporated into these tests and further studies are needed to expand upon their clinical utility.

## Exosomes

Exosomes, typically around 100 nm in diameter, are membrane-bound extracellular vesicles released by many cell types including tumor cells, that contain proteins, nucleic acids and lipids, to facilitate intercellular communication. PD-L1 is found on the surface of exosomes and exosomal PD-L1 has been linked to tumor immune evasion and development of drug resistance in cancer immunotherapy [63–65].

### Baseline levels and changes in exosomal PD-L1 associate with clinical outcome

Shimada et al. evaluated serum-derived exosomal PD-L1 in 120 patients with NSCLC (stage I-III) receiving surgical resection and reported that patients with higher exosomal PD-L1 (≥ 166 pg/mL) showed trends of worse recurrence-free survival (*p* = 0.163) [66]. However, in 17 patients in that study who experienced postoperative recurrence and were treated with anti-PD-1 therapies, those who responded to ICI had trends of elevated serum exosomal PD-L1 compared to non-responders (*p* = 0.094), and all patients with exosomal PD-L1 ≥ 166 pg/ml had disease control with anti-PD-1 therapies. Another study, by Wang et al., in advanced NSCLC patients (*n* = 149), also demonstrated that pre-treatment levels of plasma exosomal PD-L1 associated with clinical response to anti-PD-1 inhibitors [67]. However, there, patients with lower levels of exosomal PD-L1 before anti-PD-1 treatment had a higher disease control rate and longer PFS (cut-off: 0.54 pg/ml, median PFS: 238 vs. 43 days, *p* < 0.0001) than patients with levels below this threshold. Similar findings were also observed in that study in a cohort receiving ICI combination therapy.

Wang et al. also analyzed changes in plasma exosomal PD-L1 after immunotherapy and found that patients developing a clinical response had a greater increase in exosomal PD-L1 3–6 weeks post-treatment [67]. Individuals with a greater fold-change in exosomal PD-L1 demonstrated a higher disease control rate and improved PFS (*p* = 0.016 for the monotherapy group, *p* = 0.0001 for the ICI combination group) [67]. Yang and colleagues also investigated blood PD-L1 dynamics, including exosomal PD-L1, PD-L1 mRNA, and sPD-L1 levels in plasma before and after 2 months of anti-PD-(L)1 in NSCLC patients (*n* = 40) [49]. There, a greater fold change in exosomal PD-L1 (≥ 1.86) after 2 months of therapy was associated with improved OS (median 10 vs. 4.2 months, *p* = 0.011). Moreover, patients with greater changes in both blood exosomal PD-L1 and PD-L1 mRNA had a significant improvement in both PFS (*p* < 0.001) and OS (*p* < 0.001) compared to those with high changes in only one factor or with low changes in both factors. Thus, both baseline levels and dynamic changes in exosomal PD-L1 have been shown to associate with NSCLC patient outcomes following treatment with ICI.

## Circulating immune cells

Baseline levels and/or dynamic changes in the levels or phenotype of immune cells in the periphery have been reported to associate with clinical outcome in NSCLC patients following immunotherapy.

### Baseline circulating lymphocyte frequency and/or phenotype is associated with clinical outcome

Tumor infiltrating lymphocytes (TILs) play a critical role in mediating the anti-tumor effects of ICI. Given the difficulty of obtaining tumor tissues, circulating immune cells may complement and provide insights on the immune status of the tumor microenvironment, including the presence of TILs [68]. Several studies (*n* = 8) involving 634 patients have reported on the association between peripheral CD4^+^ (Table 4) and CD8^+^ (Table 5) T lymphocytes at baseline and response of NSCLC patients to ICI. Specifically, higher levels of lymphocytes, including higher percentage and count of total T cells, percentage of Ki67^+^CD8^+^ T cells and natural killer (NK) cell count prior to treatment with the anti-PD-1 inhibitor budigalimab were associated with improved clinical response and longer PFS in a phase I trial that included 40 patients with advanced NSCLC [28]. Miao et al. reported similar findings, with higher baseline CD4^+^ T cell counts with a memory phenotype (lacking expression of CD45RA, > 311.3 × 10^6^ cells/L), associating with longer PFS (*p* < 0.001) [69]. Another report, by Zhang and colleagues, in 92 stage III-IV NSCLC patients treated with durvalumab or chemoimmunotherapy found that those with higher absolute counts and percentages of CD4^+^ naïve T cells (≥ 5.5 cells/μl, ≥ 1.8%), CD4^+^ memory stem cells (≥ 64.5 cells/﻿μl, ≥ 17%) or absolute counts of CD8^+^ memory stem cells (≥ 23.5 cells/﻿μl) prior to therapy had superior PFS [70]. Notably, in this study, absolute counts of T cell subsets showed better predictive values than percentages.

**Table 4:** Association between CD4^+^ T cell subsets at baseline and clinical outcome after ICI

Biomarker		NSCLC stage (n)	Treatment	Direction at baseline	Association with clinical outcome			Ref
					**Response**	**PFS**	**OS**	
**CD4**	CD4^+^CD45RA^−^ T cell counts	IV (*n* = 136)	ICI, ICI/chemo	↑		↑ (*p* = 0.016)		[69]
	CD4^+^CD45RA^−^ T cell counts	IV (*n* = 32)	ICI	↑		↑ (*p* < 0.001)		
	CD4^+^ naïve T cell counts	III-IV (*n* = 92)	αPD-L1, αPD-L1/chemo	↑		↑ (*p* < 0.001)		[70]
	% CD4^+^ naïve T cells	III-IV (*n* = 92)	αPD-L1, αPD-L1/chemo	↑		↑ (*p* < 0.001)		
	CD4^+^ memory stem cells counts	III-IV (*n* = 92)	αPD-L1, αPD-L1/chemo	↑		↑ (*p* = 0.002)		
	% CD4^+^ memory stem cells	III-IV (*n* = 92)	αPD-L1, αPD-L1/chemo	↑		↑ (*p* = 0.018)		
	CD3^+^CD4^+^PD-1^+^	Resectable IIIA (*n* = 27)	neoadjuvant αPD-1/chemo	↑	↑ (*p* = 0.045)			[71]
	CD25 MFI on CD4^+^CD25^hi^ cells	Resectable IIIA (*n* = 27)	neoadjuvant αPD-1/chemo	↑	↑ (*p* = 0.023)			
	CD4^+^CCR9^+^	Advanced (*n* = 36)	αPD-1	↑		↓ (*p* = 0.0197)	↓ (*p* = 0.0034)	[72]
	CD4^+^CCR10^+^	Advanced (*n* = 36)	αPD-1	↑		ns	↓ (*p* = 0.0036)	

*ICI* Immune checkpoint inhibitors, *ns* Not significant, *NSCLC* Non-small cell lung cancer, *OS* Overall survival, *PFS* Progression-free survival, *MFI* Mean fluorescent intensity

**Table 5:** Association between CD8^+^ T cell subsets at baseline and clinical outcome after ICI

Biomarker		NSCLC stage (n)	Treatment	Direction at baseline	Association with clinical outcome			Ref
					**Response**	**PFS**	**OS**	
**CD8**	% Ki67^+^CD8^+^ T cells	Advanced NSCLC (*n* = 40); HNSCC (*n* = 41)	αPD-1	↑	↑ (*p* = 0.085)	↑ (*p* = 0.048)		[28]
	% CD8^+^CD45RA^−^CD62L^−^	Advanced NSCLC (*n* = 40); HNSCC (*n* = 41)	αPD-1	↑	↑ (*p* = 0.019)	ns		
	CD8^+^ memory stem cells counts	III-IV (*n* = 92)	αPD-L1, αPD-L1/chemo	↑		↑ (*p* = 0.006)		[70]
	CD8^+^CXCR4^+^	Advanced (*n* = 36)	αPD-1	↑		ns	↓ (*p* = 0.0256)	[72]
	% CD8^+^CD28^−^CD57^+^KLRG1^+^ (SIP)	III-IV (*n* = 83)	αPD-1, αPD-L1	↑	↓ (*p* = 0.002)	↓ (*p* < 0.0001)	↓ (*p* = 0.007)	[73]
	ratio of CD8^+^PD-1^+^ to CD4^+^PD-1^+^ (PERLS)	III-IV (*n* = 111)	αPD-1, αPD-L1	↑	↑ (*p* = 0.0017)	↑ (*p* = 0.0039)	↑ (*p* = 0.0239)	[74]
	SIP and PERLS	III-IV (*n* = 111)	αPD-1, αPD-L1	↓SIP, ↑PERLS		↑ (*p* < 0.0001)	↑ (*p* = 0.001)	
	CD8^+^PD-1^+^, NK cells, sPD-L1 (I_eff_S)	IIIB-IV (*n* = 109)	αPD-1, αPD-L1	no risk	↑ (*p* = 0.002)	↑ (*p* < 0.001)	↑ (*p* < 0.001)	[75]

*HNSCC* Head and neck squamous cell carcinoma, *ICI* Immune checkpoint inhibitors, *I*_*eff*_*S* Immune effector score, *NK* Natural killer, *ns* Not significant, *NSCLC* Non-small cell lung cancer, *OS* Overall survival, *PERLS* PD-1-expressing ratio of lymphocytes in a systemic blood sample, *PFS* Progression-free survival, *SIP* Senescent immune phenotype

Other studies have more extensively characterized CD4^+^ (Table 4) and CD8^+^ (Table 5) T cell subsets at baseline with additional markers and shown that cells expressing various levels of a given marker(s) associate with patient outcome. In a study of 27 stage IIIA NSCLC patients receiving neoadjuvant nivolumab plus carboplatin, Laza Briviesca et al. found that patients who subsequently developed a pathological complete response (pCR) had higher frequencies of CD3^+^CD4^+^PD-1^+^ cells, along with greater expression on a per cell basis (mean fluorescent intensity, MFI) of the NK-activating receptor NKG2D on CD3^+^CD56^+^NKG2D^+^ cells, CD56 on CD3^+^CD56^+^ cells, and CD25 on CD4^+^CD25^hi^ cells [71]. In addition, lower frequencies at baseline of CD3^−^CD56^+^CTLA-4^+^ cells and low CTLA-4 MFI on CD3^+^CD56^+^ cells were seen in this study in patients who developed pCR. In another study, Rogado et al. showed that advanced NSCLC patients (*n* = 36) with high (≥ 55 percentile) pretreatment levels of circulating CD4^+^CCR9^+^ (*p* = 0.0034), CD4^+^CCR10^+^ (*p* = 0.0036), or CD8^+^CXCR4^+^ (*p* = 0.0256) T cells had reduced OS following treatment with anti-PD-1; notably, these associations with OS were not seen in patients treated with non-immunotherapy [72].

Additional refined CD4^+^ and CD8^+^ T cell subsets and ratios of T cells have been shown to associate with patient benefit following immunotherapy. Ferrara et al. found that high circulating levels of a specific CD8^+^ T cell subset with a senescent immune phenotype (SIP, lacking expression of CD28 and expressing CD57 and KLRG1) prior to therapy associated with worse clinical response (*p* = 0.002), and reduced PFS (*p* < 0.0001) and OS (*p* = 0.007) in patients with stage III-IV NSCLC (*n* = 83) receiving anti-PD-(L)1 [73]. Of note, this association was not seen in patients who received platinum-based chemotherapy. In another study, Duchemann and colleagues reported that the ratio of CD8^+^PD-1^+^ to CD4^+^PD-1^+^ cells (termed PD-1-expressing ratio of lymphocytes in a systemic blood sample, or PERLS) prior to therapy associated with patient outcome in advanced NSCLC patients (*n* = 111) receiving anti-PD-(L)1 [74]. In that study, responding patients (with complete response (CR), PR, or SD > 6 months) had a higher PERLS ratio at baseline than non-responders (*p* = 0.0017), and patients with a high PERLS ratio (> 1.91) had a longer PFS (*p* = 0.0039) and OS (*p* = 0.0239) than patients with a lower ratio. Duchemann et al. also showed that an immunoscore consisting of SIP CD8^+^ T cells and the PERLS ratio stratified patients by good, intermediate and poor outcomes and associated with PFS (median PFS: 12.6 vs. 2.58 vs. 1.76 months, *p* < 0.0001) and OS (median OS: not reached at 14 months vs. 8.54 vs. 2.42 months, *p* = 0.001) [74]. Mazzaschi and colleagues generated a different immune score, termed the immune effector score (I_eff_S), based on pre-therapy levels of CD8^+^PD-1^+^ T cells, NK cells, and sPD-L1, to predict the efficacy of anti-PD-(L)1 inhibitors [75]. There, in a study of 109 advanced NSCLC patients receiving anti-PD-(L)1 inhibitors, patients with high numbers of CD8^+^PD1^+^ T and NK cells and low plasma sPD-L1 (classified as no risk, with a favorable I_eff_S) had a better response (*p* = 0.002), and prolonged PFS (*p* < 0.001) and OS (*p* < 0.001) than patients with at least one risk factor in the I_eff_S. The authors also reported that the predictive value of I_eff_S could be enhanced when integrated with the Lung Immune Prognostic Index (LIPI), which comprises a derived neutrophil to lymphocyte ratio (dNLR) > 3 along with LDH levels above the upper limit of normal. These studies suggest that evaluation of composite risk models based on multiple immune parameters may provide more accurate prognostic value to predict clinical benefit.

**Fig. 4:**
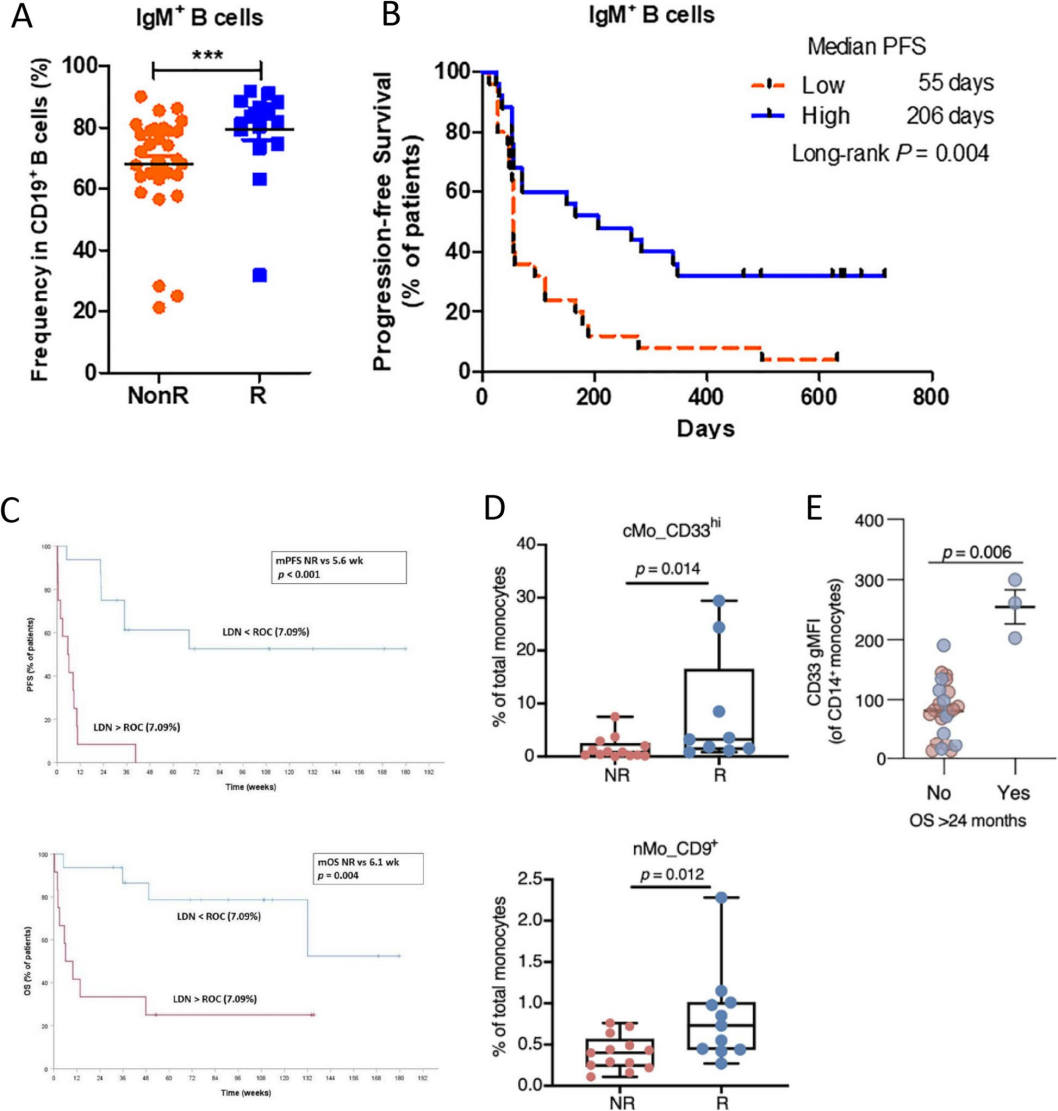
IgM^+^ B cells, low-density neutrophils, and monocytes prior to therapy associate with response of NSCLC patients to immune checkpoint inhibitors (ICI). IgM^+^ B cells prior to therapy associate with response to ICI (**A**, **B**). **A** Comparison of the percentages of IgM^+^ B cells at baseline between responder (R; *n* = 17) and non-responder (NonR; *n* = 33) NSCLC patients treated with anti-PD-1. **B** Kaplan–Meier analysis of IgM^+^ B cells at baseline with PFS. Low-density neutrophils (LDN) prior to therapy associate with response to ICI (LDNs identified as CD11b^+^CD66b^+^CD116^+^CD14^−^) (**C**). Progression-free survival (PFS; top) and overall survival (OS; bottom) stratified by the presence of baseline LDNs above the receiver operating characteristic (ROC) threshold (7.09%). Frequency of specific monocyte (Mo) subsets prior to therapy (identified through CyTOF analyses) are associated with responsiveness to anti-PD-1 (**D**, **E**). **D** Boxplots of statistically significant differences in conventional monocytes (cMo) CD33^hi^ and non-conventional monocytes (nMo) CD9^+^ that differ between non-responder (NR) and R patients. **E** Geometric mean fluorescent intensity (gMFI) of CD33 expression on cMo validated by flow cytometry in PBMCs from patients with OS > vs. < 24 months. **A**, **B** modified from Xia, ref. [77]; copyright © 2022, Frontiers. **C** modified from Arasanz, ref. [78]; MDPI open access Creative Common CC BY License. **D**, **E** modified from Olingy, ref. [79]. copyright © 2022, Frontiers

Although predictive markers to identify clinical responders following ICI have largely focused on the T cell compartment, several recent studies have interrogated other lymphocyte populations in peripheral blood for association with clinical outcome following immunotherapy (Supplemental Table 3). Lo Russo et al. evaluated the circulating immune profile of 65 patients with advanced NSCLC enrolled in a prospective phase II study of first line pembrolizumab prior to therapy and at the time of first radiological evaluation and found that abundance of the ratio of total natural killer cells/CD56^dim^CD16^+^ NK cells (*p* < 0.006) at baseline, and numbers of non-classical CD14^dim^CD16^+^monocytes (*p* = 0.0039), and eosinophils (CD15^+^CD16^−^, *p* = 0.0301) after therapy correlated with favorable PFS [76]. Another study, by Xia et al., evaluated the association between patient response and baseline levels of circulating B cells in patients with NSCLC who were treated with anti-PD-1 (*n* = 120) or anti-PD-L1 (*n* = 30) [77]. Pre-treatment percentages of total B cells (*p* = 0.004) and IgM^+^ B cells (*p* < 0.001) (Fig. 4A) were higher in responding patients compared with patients who developed PD within 180 days of anti-PD-1. Patients with higher frequencies of IgM^+^ B cells at baseline also had a longer duration of PFS than patients with lower frequencies of this subset (206 days vs. 55 days; *p* = 0.004) (Fig. 4B); of note, these findings were observed only in patients treated with anti-PD-1 as opposed to anti-PD-L1. These studies collectively demonstrate that baseline frequencies and/or counts of specific lymphocyte subsets may associate with clinical outcome following ICI. However, it should be noted that each study reviewed above focused on different populations and phenotypes of lymphocytes in interrogating whether baseline immune subsets could be associated with patient outcome. Further studies are needed to gain consensus on which specific lymphocyte populations consistently associate with patient outcome.

### Changes in lymphocyte subsets associate with clinical response

Several studies have also evaluated whether changes in peripheral CD4^+^ and CD8^+^ T lymphocytes following initiation of ICI associate with patient outcome (Supplemental Table 4). An analysis of 231 NSCLC patients treated with anti-PD-(L)1 found that high peripheral lymphocyte counts 1-month post-treatment associated with longer PFS (*p* < 0.001) and OS (*p* < 0.001) [80]. Lambert et al. also observed that 2 weeks post anti-PD-1 initiation, overall T-cell counts, CD8^+^ T-cell counts, and percentages of CD8^+^CD45RA^–^CD62L^–^ effector memory T cells were expanded in responding patients, and were associated with a longer duration of PFS (*p* = 0.024, *p* = 0.021, and *p* = 0.014, respectively) [28]. Changes in additional peripheral lymphocyte subsets have been evaluated for association with outcome in NSCLC patients treated with ICI [81, 82]. For example, Abdelfatah and colleagues evaluated the clinical utility of a different lymphocyte subset, circulating CX3CR1^+^CD8^+^ T cells, as a predictive marker of response to chemoimmunotherapy in 29 patients with stage II-IV NSCLC [82]. A > 10% increase of CX3CR1^+^CD8^+^ T cells 4 weeks after the start of treatment was associated with improved response (*p* < 0.05), PFS (*p* = 0.0051) and OS (*p* = 0.0138). The authors extended this finding with single-cell RNA and T-cell receptor (TCR) sequencing in longitudinally obtained blood samples as compared with tumor tissue and demonstrated changes in transcriptomic signatures of T cells as well as evolution of TCR clonotypes in peripheral blood containing highly frequent TIL repertoires that overexpress CX3CR1. The authors suggested that profiling T cells expressing CX3CR1 in peripheral blood not only identifies patients with improved clinical benefit, but also serves as a dynamic marker to identify T cell repertoires that are reflective of tumor-infiltrating lymphocytes.

Changes in T regulatory cells (Tregs) and B cell subsets have also been evaluated for association with patient response (Supplemental Table 4). Kang et al. reported that the frequency of total Tregs (*p* = 0.034) and PD-1^+^ Tregs (*p* < 0.001) was reduced 7 days after treatment with anti-PD-(L)1 in responders compared to non-responders, with changes in PD-1^+^ Tregs being more highly associated with response than total Tregs [83]. This study also evaluated changes in Tregs and association with development of pseudoprogression and hyperprogression, highlighting that dynamic changes in specific circulating T cell subsets may have predictive significance in NSCLC patients receiving ICI and may help to differentiate atypical responses early after the start of treatment. Xia and colleagues, who reported that high levels of IgM^+^ memory B cells prior to therapy with anti-PD-1 associated with NSCLC response, also showed that percentages of PD-1^+^ IgM^+^ memory B cells were reduced in responding patients, while percentages of PD-L1^+^ IgM^+^ memory B cells were increased in responding patients [77]. These studies highlight that changes in specific peripheral lymphocyte subsets, including T cells, Tregs, or B cells, can associate with clinical outcome following ICI in NSCLC patients, and may have prognostic value.

### Higher baseline NLR and increases after treatment are associated with poor clinical outcome

Many studies have evaluated the association between clinical response of NSCLC patients to ICI and the neutrophil-to-lymphocyte ratio (NLR), an easily obtained blood-based parameter that reflects the inflammation status of a patient, with both single studies, meta analyses, and comprehensive reviews reporting a negative association with outcome [57, 84–90]. As an example, a meta-analysis by Li et al. of > 2,000 NSCLC patients receiving anti-PD-(L)1 showed that a high pretreatment NLR was associated with inferior PFS (*p* < 0.001) and OS (*p* < 0.001) [87]. Dynamic changes in the NLR were also assessed, with an increase in the NLR (≥ 3.0) 6 weeks after anti-PD-(L)1 associating with poor PFS (median: 3.1 vs. 9.1 months, *p* = 0.01) and OS (median: 6.8 vs. 17.0 months, *p* < 0.0001). These findings of the NLR serving as a poor prognostic indicator have been combined with other immune parameters to develop highly predictive models of response. Hwang et al., using machine learning to integrate clinical characteristics and peripheral immune cell dynamics in 239 patients with metastatic NSCLC treated with anti-PD-1 or anti-CTLA-4, identified that a low NLR and high eosinophil fraction at the time of the first radiographic follow-up, low NLR at 4 weeks, and the relative change in NLR at these time points compared with baseline were the strongest predictors of clinical benefit [91]. The authors extended these findings in a cohort of NSCLC patients treated with neoadjuvant ICI and found that NLR dynamics were predictive of both pathologic response and duration of recurrence-free survival (*p* = 0.0097). These studies collectively demonstrate that high baseline levels of the NLR and increases in the NLR after ICI associate with poor prognosis of NSCLC patients.

### Higher eosinophil counts at baseline and increase after therapy are associated with improved clinical outcome

Two studies representing 324 patients have shown that baseline levels of circulating eosinophils, an immune subset easily obtained through analysis of complete blood counts and often overlooked in the context of immunotherapy, associate with response of NSCLC patients to ICI [91–94] (Supplemental Table 5). In advanced or metastatic NSCLC patients (*n* = 166) treated with anti-PD-(L)1, Takeuchi et al. reported that patients with an intermediate pre-treatment eosinophil count (between 100–500 cells/μl) displayed a prolonged OS (*p* < 0.001) compared with patients with levels below or above this range [92]. Caliman et al. reported that high absolute eosinophil counts prior to therapy (≥ 130 cells/μl) correlated with an improved response rate (64.4% vs. 35.6%, *p* = 0.009), and longer PFS (*p* = 0.007, 7.0 vs. 2.5 months) and OS (*p* = 0.009, 9.0 vs. 5.5 months) in 158 stage IIIB-IV NSCLC patients [94]. In another study of 53 NSCLC patients treated anti-PD-1, patients with high eosinophil counts (> 90 cells/μl) at baseline, along with high counts of granulocytic myeloid-derived suppressor cells (Gr-MDSC, ≥ 6 cells/μl), low neutrophil count (< 5840 cells/μl), and low NLR (< 3), were classified as having a “good immune-asset”; patients with this combined phenotype exhibited an improvement in both PFS (*p* = 0.015) and OS (*p* = 0.05) [93]. One study reported on the relationship between changes in eosinophil counts and response of NSCLC patients to ICI, with increases after 4 weeks of neoadjuvant ICI associating to tumor regression (*p* = 0.0086) and major pathologic response (*p* = 0.005) [91] (Supplemental Table 6). These data collectively suggest that high eosinophil counts, both prior to and after therapy, associate with improved outcomes in NSCLC patients receiving ICI.

**Table 6:** Association between monocyte subsets at baseline and clinical outcome after ICI

Biomarker		NSCLC stage (n)	Treatment	Direction at baseline	Association with clinical outcome			Ref
					**Response**	**PFS**	**OS**	
**Monocyte**	CD69 MFI on intermediate monocytes	Resectable IIIA (*n* = 27)	Neoadjuvant αPD-1/chemo	↑	↑ (*p* = 0.017)			[71]
	% CD14^++^CD16^+^CTLA4^+^	Resectable IIIA (*n* = 27)	neoadjuvant αPD-1/chemo	↓	↑ (*p* = 0.026)			
	CD33^hi^ monocytes	III-IV (*n* = 26)	αPD-1	↑	↑ (*p* = 0.014)			[79]
	CD9^+^ non-classical monocytes	III-IV (*n* = 26)	αPD-1	↑	↑ (*p* = 0.012)			
	CD33 gMFI on CD14^+^ monocytes	III-IV (*n* = 26)	αPD-1	↑	↑ (*p* = 0.075)		↑ (*p* = 0.006)	
	PD-L1^+^ CD14^+^ monocytes	Advanced (*n* = 119)	αPD-1, αPD-L1, αPD-1/chemo, αPD-L1/chemo	↑		↑ (*p* < 0.001)	↑ (*p* = 0.02)	[95]

*gMFI *geometric mean fluorescent intensity*, ICI* Immune checkpoint inhibitors, *MFI* Mean fluorescent intensity, *NSCLC* Non-small cell lung cancer, *OS* Overall survival, *PFS* Progression-free survival

### Association between low density neutrophils, monocytes and platelets and patient response

Baseline peripheral levels of low-density neutrophils have also been evaluated for association with response in NSCLC patients receiving ICI. Arasanz and colleagues, using flow cytometry, identified a population of low-density neutrophils (LDN, defined as CD11b^+^CD116^+^CD66b^+^CD3^−^CD14^−^) in peripheral blood of 31 patients with NSCLC prior to treatment with frontline pembrolizumab that associated inversely with outcome [78]. An LDN threshold > 7.09% identified patients with disease control < 6 months with a high sensitivity (84.6%) and specificity (93.3%), with patients above this threshold having a shorter duration of PFS (*p* < 0.001) and OS (*p* = 0.004) (Fig. 4C).

Monocyte subsets have emerged as a population of interest as a potential biomarker of response to ICI [96]. Multiple studies (*n* = 3) involving 172 NSCLC patients have found that high peripheral monocyte populations at baseline associate with improved response rates, and in some cases improved PFS and OS (Table 6). Specifically, Olingy and colleagues, in 26 stage III-IV NSCLC patients treated with anti-PD-1, evaluated the association between monocyte subsets (identified by mass cytometry) and clinical response [79]. Responders (R, with a decreased tumor size or SD > 6 months) had greater baseline frequencies of CD33^hi^ classical monocytes (CD33^hi^ cMo, *p* = 0.014) and CD9^+^ nonclassical monocytes (CD9^+^ nMo, *p* = 0.012) than non-responding (NR) patients (Fig. 4D). The importance of CD33 expression on cMo was confirmed by flow cytometry, with the authors showing that the degree of CD33 expression on CD14^+^ monocytes prior to therapy associated with OS (*p* < 0.006) (Fig. 4E). Laza-Briviesca et al., in a study of 27 stage IIIA NSCLC patients receiving neoadjuvant nivolumab plus carboplatin, found that patients who subsequently developed a pCR had greater expression on a per cell basis (MFI) of CD69 on intermediate monocytes, and lower frequencies of CD14^++^CD16^+^CTLA-4^+^ cells than patients who did not develop pCR [71]. Another study, by Zamora Atenza and colleagues, in 119 patients with advanced NSCLC treated with anti-PD-(L)1, sought to integrate PD-L1 expression on various immune subsets in peripheral blood, including monocytes and platelets, as predictive biomarkers of response [95]. Patients with high baseline frequencies of PD-L1^+^ CD14^+^ monocytes, PD-L1^+^ neutrophils, and PD-L1^+^ platelets displayed longer periods of PFS, with PD-L1^+^ monocytes showing the strongest association (*p* < 0.001); prolonged OS (*p* = 0.02) was also observed in patients with higher percentages of PD-L1^+^ monocytes. In their study, Hinterleitner and colleagues evaluated the association between PD-L1^+^ platelets and clinical response to ICI [97]. The authors devised a matrix termed pPD-L1^Adj^ based on levels of platelet PD-L1^+^ and the platelet activation marker CD62P. Patients with a high pPD-L1^Adj^ had superior PFS following treatment with anti-PD-1 (*p* = 0.003), but inferior PFS in a different cohort of patients treated with conventional chemotherapy. These studies collectively show promising associations between pre-therapy frequencies of circulating monocytes and platelets, including those that express PD-L1, as a potential biomarker of response of NSCLC patients to ICI.

## Cell-free DNA and blood TMB

### Low baseline levels of cfDNA and decrease in cfDNA after therapy associate with better outcomes

Cell-free DNA (cfDNA) consists of small double-stranded DNA fragments (typically < 200 base pairs) that are released into the bloodstream from cells undergoing apoptosis, necrosis, or other cellular turnover. These fragments are detectable in serum or plasma, and in cancer patients, some fraction reflects circulating tumor DNA (ctDNA), and can mirror the genetic and epigenetic modifications of the tissue of origin [98–100]. Alama et al. analyzed total cfDNA levels prior to nivolumab treatment in 89 advanced NSCLC patients for association with response and found that patients with cfDNA concentrations below the median (836.5 ng/3mL plasma) had a longer OS (*p* = 0.04) than patients with higher cfDNA [101].

**Fig. 5:**
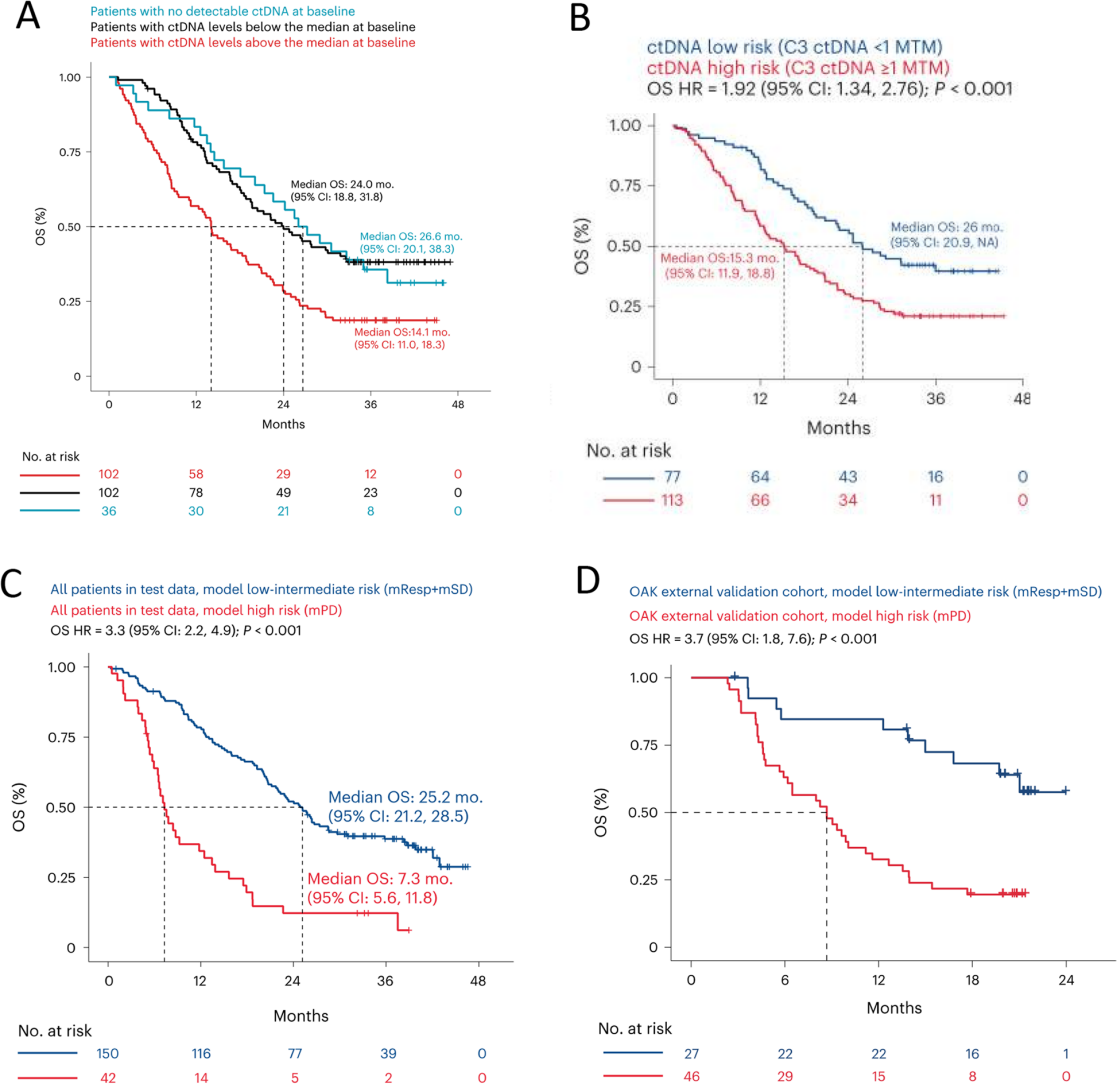
On treatment ctDNA dynamics associate with survival benefit. Kaplan Meier (KM) curves of overall survival (OS) for patients in a training set (*n* = 240) with ctDNA levels at baseline in patients ctDNA negative (blue, zero mutations detected), ctDNA levels ≥ 64 mean tumor molecules (MTM; median, red), and < 64 MTM (black) (**A**). KM curves of OS for patients in a training set (*n* = 190) with ctDNA levels at C3D1 below the assay limit of detection (LOD; < 1 MTM, ctDNA low risk, blue) versus ≥ 1 MTM (ctDNA high risk, red) (**B**). A machine learning model was built in the training set based on 5 ctDNA features (MTM at C3D1, # of pathogenic mutations at C3D1, change in # of mutations detected from baseline to C2D1, total cell-free DNA concentration at C3D1, and area under the curve (AUC) for ctDNA level from baseline to C2D1). This model predicted the stratification of patients by molecular risk that associated with OS in a hold-back test set (*n* = 192) (**C**) and an external validation cohort (OAK, *n* = 73) (**D**). Patients in (**C**) and (**D**) predicted to be in the high-risk group (mPD, red) have a worse OS than patients predicted to have a molecular response or stable disease (mResp + mSD, blue). Modified from Assaf, ref. [104].; copyright © 2023, Springer Nature

More studies, including four detailed in Table 7 involving > 1,800 NSCLC patients treated with ICI, have evaluated the prognostic potential of ctDNA, with baseline levels in some cases, and post-treatment levels in all cases, associating with response [102–105]. In a meta-analysis of 1,017 NSCLC patients from 10 studies, Wang et al. found that pre-treatment ctDNA levels did not associate with clinical response to ICI; however, reduction in ctDNA after ICI strongly associated with improved ORR (*p* < 0.001), PFS (*p* < 0.001), and OS (*p* < 0.001) [105]. In another study, Assaf et al. longitudinally monitored ctDNA at five timepoints in stage IV NSCLC patients treated with anti-PD-L1 combined with chemotherapy and built a machine learning model integrating multiple ctDNA metrics to predict OS [104]. In a training set, patients at baseline with ctDNA above the median had shorter OS than patients with ctDNA levels below the median (*p* < 0.001) (Fig. 5A). In addition, patients with ctDNA levels at cycle 3 day 1 below the limit of detection (LOD) had superior OS compared with patients with levels at or above the LOD (*p* < 0.001) (Fig. 5B). The authors then generated a model comprising five ctDNA features that discriminated high-risk from low-intermediate-risk patients by OS. This association was shown in both a hold back test set (*n* = 192, median OS 7.3 vs. 25.2 months for high-risk vs. low-intermediate-risk patients, *p* < 0.001) (Fig. 5C), and an external validation cohort (OAK, *n* = 73, *p* < 0.001) (Fig. 5D). These studies collectively support the predictive value of measuring ctDNA in NSCLC patients throughout the course of ICI to assist in determining potential long-term clinical benefit.

**Table 7:** Association between ctDNA before and after ICI and clinical outcome

Biomarker		NSCLC stage (n)	Treatment	Direction	Association with clinical outcome			Ref
					**Response**	**PFS**	**OS**	
**Baseline**	ctDNA	III-IV (*n* = 10 studies)	ICI based	↓	ns	ns	ns	[105]
		IV nonsquamous (*n* = 446)	αPD-L1/bev/chemo, αPD-L1/chemo, bev/chemo	↓		↑ (*p* = 0.002)	↑ (*p* < 0.001)	[104]
		IV squamous (*n* = 221)	αPD-L1/chemo, chemo	↓		↑	↑	[103]
**Post treatment**	ctDNA	III-IV (*n* = 10 studies)	ICI based	↓	↑ (*p* < 0.001)	↑ (*p* < 0.001)	↑ (*p* < 0.001)	[105]
		IV nonsquamous (*n* = 446)	αPD-L1/bev/chemo, αPD-L1/chemo, bev/chemo	↓ (model)			↑ (*p* < 0.001)	[104]
		IV squamous (*n* = 221)	αPD-L1/chemo, chemo	↓	↑	↑ (*p* < 0.001)	↑ (*p* < 0.001)	[103]
		III-IV squamous (*n* = 134)	αPD-1/chemo	↓	↑ (*p* = 0.004)	↑ (*p* < 0.001)	↑ (*p* < 0.001)	[102]

*ctDNA* Circulating tumor DNA, *ICI* Immune checkpoint inhibitors, *ns* Not significant, *NSCLC* Non-small cell lung cancer, *OS* Overall survival, *PFS* Progression-free survival

### High blood tumor mutational burden (bTMB) predicts a favorable response to immunotherapy

TMB is a tissue-based biomarker that has been extensively explored to predict clinical outcomes of cancer patients to ICI [16]. Gandara et al. developed a blood-based assay to measure TMB in plasma (termed bTMB) [106], which has been assessed at baseline in multiple other studies [107–115] for its association with patient response (Supplemental Table 7). In their study, bTMB positively correlated with tissue-based TMB [106], and the authors found that patients with NSCLC treated with atezolizumab had improved PFS compared with patients treated with docetaxel in a training cohort (POPLAR, *n* = 211, *p* = 0.055) and confirmatory cohort (OAK, *n* = 583, *p* = 0.036). As another example, Kim et al. conducted a prospective study evaluating bTMB in patients with advanced NSCLC treated with atezolizumab in the first-line; there bTMB ≥ 16 was associated with both a higher ORR (*p* < 0.0001) and longer OS (median OS: 29.1 vs. 13.4 months, *p* = 0.032) [107]. Based on this study, and the findings of many others also reporting a positive association between bTMB and response to immunotherapy [108–115], the use of plasma as a DNA source in which to determine tumor mutation burden makes bTMB an attractive option for those patients who may not be amenable to biopsy or those for whom tumor tissue is unavailable.

## Epigenetic regulation

Epigenetics regulate gene expression without changing DNA sequence, by altering chromatin structure, nuclear organization, and transcript stability. Epigenetics are well known to play a vital role in the occurrence and development of many cancers, including NSCLC, and epigenetic regulation of gene expression occurs at three levels: DNA methylation, histone/nucleosome modification, and non-coding RNA (ncRNA) [116, 117]. Changes in DNA methylation can be measured in peripheral blood, and extensive studies in NSCLC have reported on blood-based DNA methylation as a biomarker in relation to the risk of developing lung cancer and/or early detection of disease [118, 119]. Histone/nucleosome modifications, including acetylation and methylation, can also be measured in peripheral blood [120–122]; however, no studies to date have reported on the association between clinical response of NSCLC patients to ICI and either blood-based DNA methylation patterns or histone modifications. ncRNAs are RNA molecules that are not translated into proteins and include short (< 200 nucleotides) and long (> 200 nucleotides) ncRNAs and can be detected in diverse bodily fluids including blood. miRNAs are short ncRNAs (~ 22 nucleotides) that bind to complementary sequences in the 3′ untranslated region (UTR) of target mRNAs to provide post-transcriptional regulation of protein-coding genes. Depending on the degree of miRNA/mRNA complementarity, this interaction leads to mRNA repression or degradation, and inhibition of translation.

### Expression of circulating miRNAs associate with clinical response

Circulating miRNA profiles have been extensively studied in NSCLC patients in relation to clinical outcomes, with elevated levels of certain circulating miRNAs and reduced expression of others associating with the clinical outcome of NSCLC patients treated with ICIs [123–130]. Monastirioti et al., for example, conducted an analysis on the pre-treatment levels and clinical relevance of multiple immunoregulatory miRNAs implicated in immune checkpoint regulation (miR-34a, miR-200b, miR-200c), T-cell activity (miR-155), the function of MDSCs (miR-223), and the function of Tregs (miR-146a) in a cohort of 69 advanced NSCLC patients treated with second-/third-line nivolumab [128]. Patients with lower baseline plasma miR-200c levels exhibited notably extended OS (*p* = 0.003), along with trends towards longer PFS (*p* = 0.146). In subgroup analyses low miR-200c and high miR-34a levels correlated with improved response rates and prolonged PFS and OS in non-squamous patients, while elevated expression of miR-146a and miR-223 associated with prolonged disease control duration in squamous patients. In another study, Rajakumar and colleagues conducted whole blood miRNA profiling in 334 stage IV NSCLC patients and established a 5 microRNA risk score (miRisk), comprising miR-2115-3p, miR-218-5p, miR-244-5p, miR-4676-3p, and miR-6503-5p, capable of predicting overall survival following immunotherapy [129]. Patients categorized in the low-risk group exhibited significantly prolonged OS compared to those in the high-risk group across both training (*n* = 96, *p* < 0.001) and validation (*n* = 99, *p* = 0.0021) cohorts treated with anti-PD-1 monotherapy. This distinction was notably not observed in patients treated with chemoimmunotherapy (*n* = 139). These studies [123–130] and others collectively affirm the potential of altered circulating miRNAs as viable biomarkers for predicting clinical response of NSCLC patients to ICI.

## Circulating tumor cells

### High baseline CTCs and increase in CTC during treatment are associated with worse outcome

Circulating tumor cells (CTCs) are tumor cells derived from primary or metastatic lesions that travel into the bloodstream and may reflect the biologic characteristics of the tumor from which they originate [131, 132]. CTCs are present at baseline in 32%—93% of patients with NSCLC, and several studies have reported that NSCLC patients with high values at baseline have worse clinical outcomes following ICI [101, 133, 134] (Supplemental Table 7). Alama et al. reported a four-fold risk of death in patients treated with nivolumab who had both CTCs and cfDNA concentrations above median values (*p* < 0.001) [101]. In another study, Tamminga and colleagues reported that high baseline CTC values in 104 stage IIIB-IV NSCLC patients associated with both PFS (*p* < 0.01) and OS (*p* < 0.01) [133]. This study also found that changes in CTC number after immunotherapy associated with response; patients with increases or stable levels in CTCs 4 weeks post-ICI had a lower durable response rate (*p* = 0.04), and worse PFS (*p* = 0.04) and OS (*p* = 0.04) than patients with reductions in CTCs [133] (Supplemental Table 8).

Given that PD-L1 is a dynamic marker that can be regulated by therapy, several studies have explored PD-L1 expression on CTC as a potential prognostic factor (Supplemental Tables 7 and 8). Several studies have shown that baseline PD-L1 expression on CTC was not correlated with tissue PD-L1 [134, 135]; however, higher frequencies of PD-L1^+^ CTCs (≥ 1%) prior to nivolumab treatment were observed in patients with shorter PFS (< 6 months), and PD-L1^+^ CTCs were present at the time of progression in all individuals who progressed after treatment with nivolumab [134]. In line with these findings, in a small study of 11 patients with advanced NSCLC, Janning and colleagues found that all CTCs were PD-L1 positive upon development of resistance to anti-PD-(L)1 treatment, and that all responding patients had a decrease or no change in PD-L1^+^ CTC relative to baseline at the time of response (*p* = 0.001) [135]. Several limitations to using CTC as a predictive biomarker exist, including a lack of consensus on the methods used to isolate and measure CTCs, and the cut points to determine high versus low levels. Larger studies are needed to ascertain the role of CTCs as a biomarker of response in NSCLC patients treated with immunotherapy.

## Concluding remarks

Interrogation of peripheral blood holds enormous potential in identifying immune correlates of clinical response, especially in the setting of solid tumors, wherein the primary tumor site is often not easily accessible and challenging to profile at multiple time points throughout the course of therapy. Circulating levels of IL-6, IL-8, NLR, and ctDNA, for example, both pre- and post-treatment with ICI showed impressive association with clinical responses. While these blood-based assays present a promising avenue for assessing treatment response and clinical outcomes, it should be noted that there are some limitations. When interpreting data, it should be remembered that levels of circulating soluble analytes and immune subsets may be influenced by factors such as prior/concurrent infection and other medications. One may also encounter difficulties in establishing consistent predictive values for these measures, as many studies use different methodologies and different thresholds/cutoffs. ctDNA and bTMB, though indicative of tumor burden and mutations, suffer from detection variability among platforms and lack standardized thresholds for clinical significance. In addition, CTCs, while providing insights into tumor biology and metastatic potential, face technical challenges due to their rarity in blood and variability in isolation methods. Isolation of exosomes also differs greatly among laboratories, which may result in inconsistent and variable populations. Circulating miRNAs exhibit promise in identifying associations with immunotherapy response in NSCLC patients; however, standardized protocols and understanding of their regulatory complexities and determining which of many to evaluate are needed in order for them to serve as reliable and predictive biomarkers.

Despite these obstacles, combining multiple assays in peripheral blood or integrating them with traditional clinical factors, each of which holds its own promise in identifying correlates of clinical response, has the potential to improve the ability of clinicians to identify those patients who may best respond to a given therapy. The vast majority of the studies reviewed here have evaluated only one or two of these types of assays, and more comprehensive analyses are warranted. Bioinformatic analyses of peripherally derived signatures may allow for more opportunities to tailor treatment to maximize patient benefit in the rapidly evolving field of cancer immunotherapy. Future advancements, standardization of techniques, and larger scale validation studies are imperative to harness the full potential of these blood-based biopsy methods for personalized treatment strategies in NSCLC patients receiving immunotherapy. In some cases, studies on blood-based biomarkers have reported on how a particular peripheral measure compares with tissue approved biomarkers (e.g., PD-L1 expression and TMB) that are used for clinical decisions. More of these comparative studies are needed in this rapidly growing field. It should be noted that, while this review focused on promising advances in the field of blood-based biomarkers in NSCLC patients treated with ICI, the blood-based assays described here are not cancer specific and could be considered more widely for application in immunotherapy studies of all solid tumors. In closing, this review demonstrates the potential value that specific blood-based biomarkers have provided in predicting therapeutic responses and long-term outcomes of patients following immune checkpoint inhibitors.

## Supplementary Information

**Additional file 1: Supplemental Table 1.** Association between soluble factors after ICI therapy and clinical outcome. **Supplemental Table 2.** Association between soluble factors and proteomic tests at baseline and clinical outcome after ICI. **Supplemental Table 3.** Association between circulating lymphoid immune cells at baseline and clinical outcome after ICI. **Supplemental Table 4.** Association between circulating lymphoid cells after ICI therapy and clinical outcome. **Supplemental Table 5.** Association between circulating myeloid immune cells at baseline and clinical outcome after ICI. **Supplemental Table 6.** Association between circulating myeloid cells after ICI therapy and clinical outcome. **Supplemental Table 7.** Association between circulating DNA, blood tumor mutation burden, and circulating tumor cells at baseline and clinical outcome after ICI. **Supplemental Table 8.** Association between CTCs after ICI therapy and clinical outcome.

## Data Availability

All data and materials are included in the references.

## Abbreviations

PD-1: Programmed death 1
PD-L1: Programmed death-ligand 1
NSCLC: Non-small cell lung cancer
ICI: Immune checkpoint inhibitors
ORR: Objective response rate
TMB: Tumor mutational burden
cfDNA: Cell-free DNA
ctDNA: Circulating tumor DNA
bTMB: Blood-based tumor mutational burden
CTC: Circulating tumor cells
IL-6: Interleukin 6
PFS: Progression-free survival
HNSCC: Head and neck squamous cell carcinoma
CRP: C-reactive protein
OS: Overall survival
BOR: Best overall response
PD: Progressive disease
SD: Stable disease
PR: Partial response
IL-8: Interleukin 8
HIC: Host immune classifier
HIC-H: HIC-hot
HIC-C: HIC-cold
PIR: Primary immune response
TIL: Tumor infiltrating lymphocytes
pCR: Pathological complete response
MFI: Mean fluorescent intensity
TCR: T-cell receptor
Tregs: T regulatory cells
NLR: Neutrophil-to-lymphocyte ratio
LDN: Low-density neutrophil
NonR/NR: Non-responder
