# Supplementary material for: Blood-based biomarkers in patients with non-small cell lung cancer treated with immune checkpoint blockade
Source: J Exp Clin Cancer Res. 2024 Mar 16;43:82. doi: 10.1186/s13046-024-02969-1 (PMC10944611; doi:10.1186/s13046-024-02969-1)
Supplement: Supplementary file 1 — Additional file 1: Supplemental Table 1. Association between soluble factors after ICI therapy and clinical outcome. Supplemental Table 2. Association between soluble factors and proteomic tests at baseline and clinical outcome after ICI. Supplemental Table 3. Association between circulating lymphoid immune cells at baseline and clinical outcome after ICI. Supplemental Table 4. Association between circulating lymphoid cells after ICI therapy and clinical outcome. Supplemental Table 5. Association between circulating myeloid immune cells at baseline and clinical outcome after ICI. Supplemental Table 6. Association between circulating myeloid cells after ICI therapy and clinical outcome. Supplemental Table 7. Association between circulating DNA, blood tumor mutation burden, and circulating tumor cells at baseline and clinical outcome after ICI. Supplemental Table 8. Association between CTCs after ICI therapy and clinical outcome. [file 13046_2024_2969_MOESM1_ESM.pptx]

## Slide 1
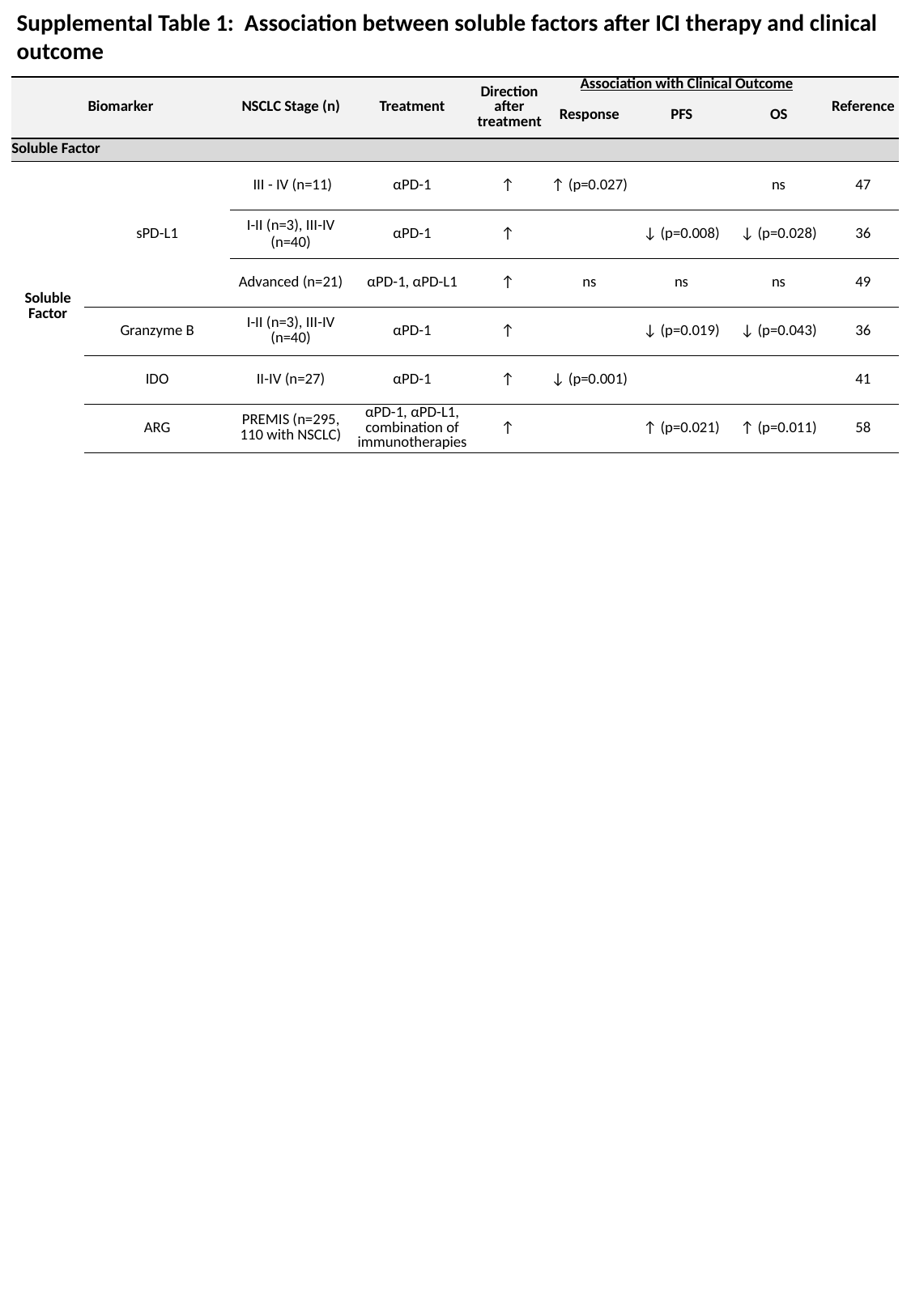

Supplemental Table 1: Association between soluble factors after ICI therapy and clinical outcome
| Biomarker | | NSCLC Stage (n) | Treatment | Direction after treatment | Association with Clinical Outcome | | | Reference |
| --- | --- | --- | --- | --- | --- | --- | --- | --- |
| | | | | | Response | PFS | OS | |
| Soluble Factor | | | | | | | | |
| Soluble Factor | sPD-L1 | III - IV (n=11) | αPD-1 | ↑ | ↑ (p=0.027) | | ns | 47 |
| | | I-II (n=3), III-IV (n=40) | αPD-1 | ↑ | | ↓ (p=0.008) | ↓ (p=0.028) | 36 |
| | | Advanced (n=21) | αPD-1, αPD-L1 | ↑ | ns | ns | ns | 49 |
| Soluble Factor | Granzyme B | I-II (n=3), III-IV (n=40) | αPD-1 | ↑ | | ↓ (p=0.019) | ↓ (p=0.043) | 36 |
| | IDO | II-IV (n=27) | αPD-1 | ↑ | ↓ (p=0.001) | | | 41 |
| | ARG | PREMIS (n=295, 110 with NSCLC) | αPD-1, αPD-L1, combination of immunotherapies | ↑ | | ↑ (p=0.021) | ↑ (p=0.011) | 58 |

## Slide 2
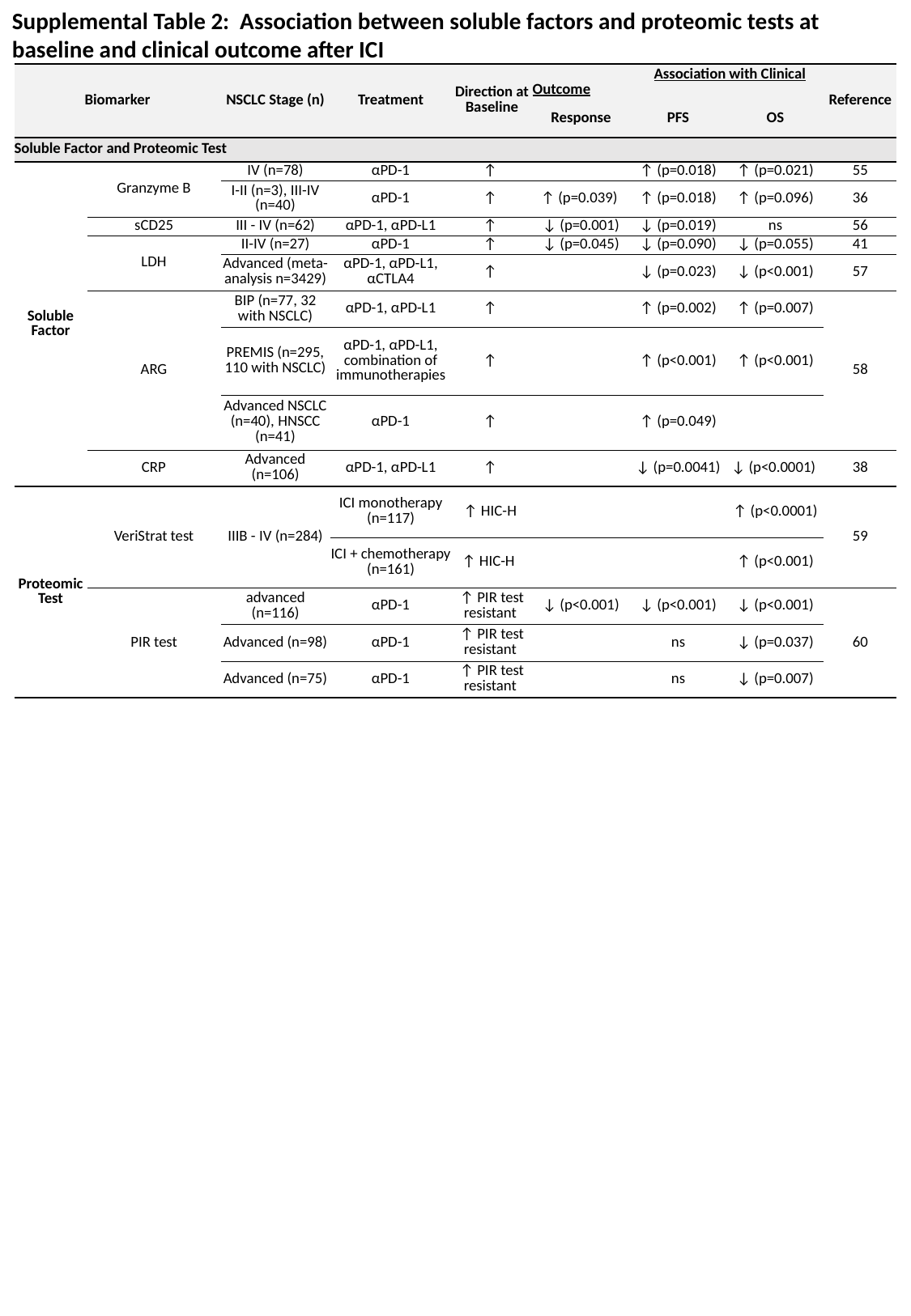

Supplemental Table 2: Association between soluble factors and proteomic tests at baseline and clinical outcome after ICI
| Biomarker | | NSCLC Stage (n) | Treatment | Direction at Baseline | Association with Clinical Outcome | | | Reference |
| --- | --- | --- | --- | --- | --- | --- | --- | --- |
| | | | | | Response | PFS | OS | |
| Soluble Factor and Proteomic Test | | | | | | | | |
| Soluble Factor | Granzyme B | IV (n=78) | αPD-1 | ↑ | | ↑ (p=0.018) | ↑ (p=0.021) | 55 |
| | | I-II (n=3), III-IV (n=40) | αPD-1 | ↑ | ↑ (p=0.039) | ↑ (p=0.018) | ↑ (p=0.096) | 36 |
| | sCD25 | III - IV (n=62) | αPD-1, αPD-L1 | ↑ | ↓ (p=0.001) | ↓ (p=0.019) | ns | 56 |
| | LDH | II-IV (n=27) | αPD-1 | ↑ | ↓ (p=0.045) | ↓ (p=0.090) | ↓ (p=0.055) | 41 |
| | | Advanced (meta-analysis n=3429) | αPD-1, αPD-L1, αCTLA4 | ↑ | | ↓ (p=0.023) | ↓ (p<0.001) | 57 |
| | ARG | BIP (n=77, 32 with NSCLC) | αPD-1, αPD-L1 | ↑ | | ↑ (p=0.002) | ↑ (p=0.007) | 58 |
| | | PREMIS (n=295, 110 with NSCLC) | αPD-1, αPD-L1, combination of immunotherapies | ↑ | | ↑ (p<0.001) | ↑ (p<0.001) | |
| | | Advanced NSCLC (n=40), HNSCC (n=41) | αPD-1 | ↑ | | ↑ (p=0.049) | | |
| | CRP | Advanced (n=106) | αPD-1, αPD-L1 | ↑ | | ↓ (p=0.0041) | ↓ (p<0.0001) | 38 |
| Proteomic Test | VeriStrat test | IIIB - IV (n=284) | ICI monotherapy (n=117) | ↑ HIC-H | | | ↑ (p<0.0001) | 59 |
| | | | ICI + chemotherapy (n=161) | ↑ HIC-H | | | ↑ (p<0.001) | |
| | PIR test | advanced (n=116) | αPD-1 | ↑ PIR test resistant | ↓ (p<0.001) | ↓ (p<0.001) | ↓ (p<0.001) | 60 |
| | | Advanced (n=98) | αPD-1 | ↑ PIR test resistant | | ns | ↓ (p=0.037) | |
| | | Advanced (n=75) | αPD-1 | ↑ PIR test resistant | | ns | ↓ (p=0.007) | |

## Slide 3
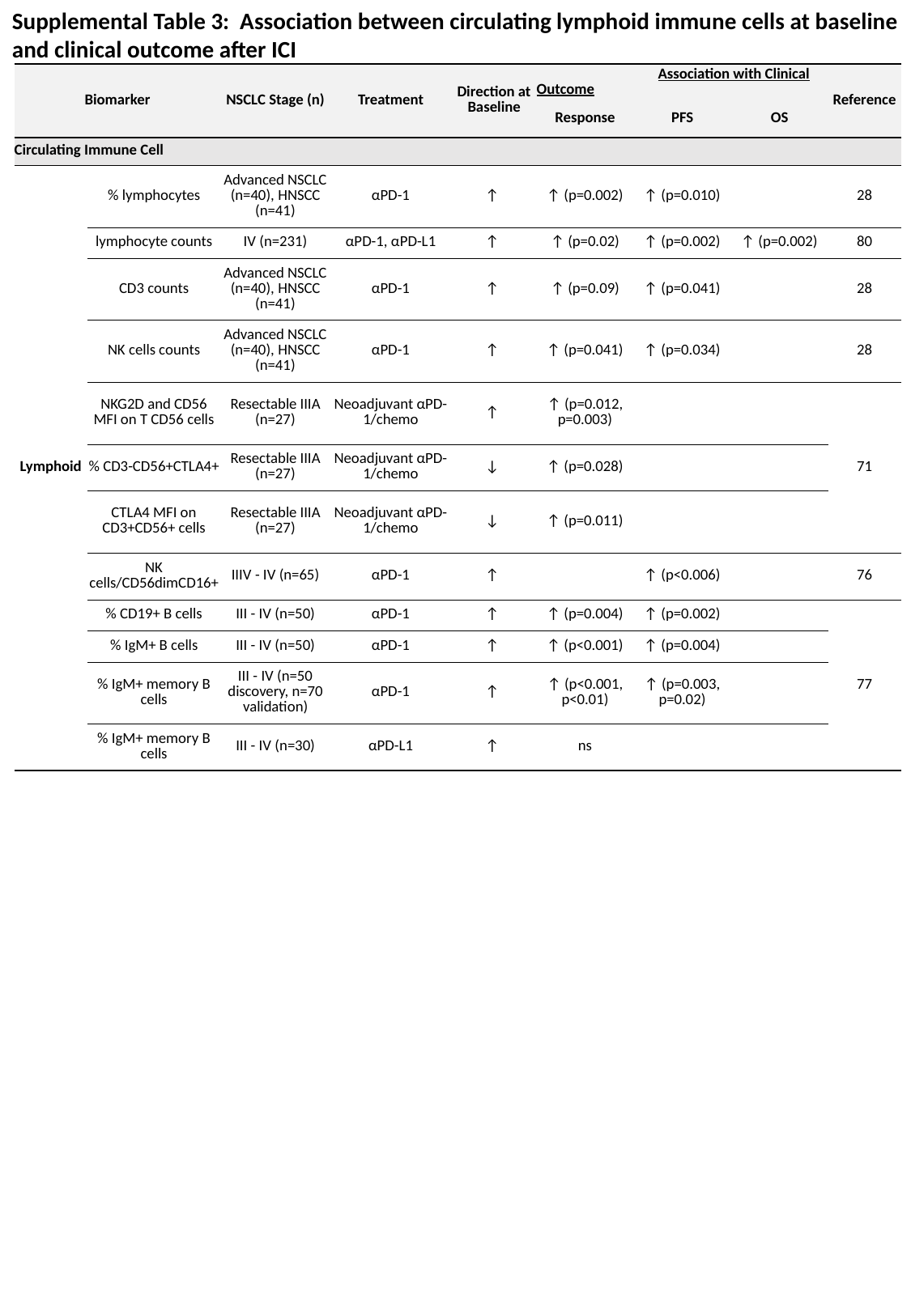

Supplemental Table 3: Association between circulating lymphoid immune cells at baseline and clinical outcome after ICI
| Biomarker | | NSCLC Stage (n) | Treatment | Direction at Baseline | Association with Clinical Outcome | | | Reference |
| --- | --- | --- | --- | --- | --- | --- | --- | --- |
| | | | | | Response | PFS | OS | |
| Circulating Immune Cell | | | | | | | | |
| Lymphoid | % lymphocytes | Advanced NSCLC (n=40), HNSCC (n=41) | αPD-1 | ↑ | ↑ (p=0.002) | ↑ (p=0.010) | | 28 |
| | lymphocyte counts | IV (n=231) | αPD-1, αPD-L1 | ↑ | ↑ (p=0.02) | ↑ (p=0.002) | ↑ (p=0.002) | 80 |
| | CD3 counts | Advanced NSCLC (n=40), HNSCC (n=41) | αPD-1 | ↑ | ↑ (p=0.09) | ↑ (p=0.041) | | 28 |
| | NK cells counts | Advanced NSCLC (n=40), HNSCC (n=41) | αPD-1 | ↑ | ↑ (p=0.041) | ↑ (p=0.034) | | 28 |
| | NKG2D and CD56 MFI on T CD56 cells | Resectable IIIA (n=27) | Neoadjuvant αPD-1/chemo | ↑ | ↑ (p=0.012, p=0.003) | | | 71 |
| | % CD3-CD56+CTLA4+ | Resectable IIIA (n=27) | Neoadjuvant αPD-1/chemo | ↓ | ↑ (p=0.028) | | | |
| | CTLA4 MFI on CD3+CD56+ cells | Resectable IIIA (n=27) | Neoadjuvant αPD-1/chemo | ↓ | ↑ (p=0.011) | | | |
| | NK cells/CD56dimCD16+ | IIIV - IV (n=65) | αPD-1 | ↑ | | ↑ (p<0.006) | | 76 |
| | % CD19+ B cells | III - IV (n=50) | αPD-1 | ↑ | ↑ (p=0.004) | ↑ (p=0.002) | | 77 |
| | % IgM+ B cells | III - IV (n=50) | αPD-1 | ↑ | ↑ (p<0.001) | ↑ (p=0.004) | | |
| | % IgM+ memory B cells | III - IV (n=50 discovery, n=70 validation) | αPD-1 | ↑ | ↑ (p<0.001, p<0.01) | ↑ (p=0.003, p=0.02) | | |
| | % IgM+ memory B cells | III - IV (n=30) | αPD-L1 | ↑ | ns | | | |

## Slide 4
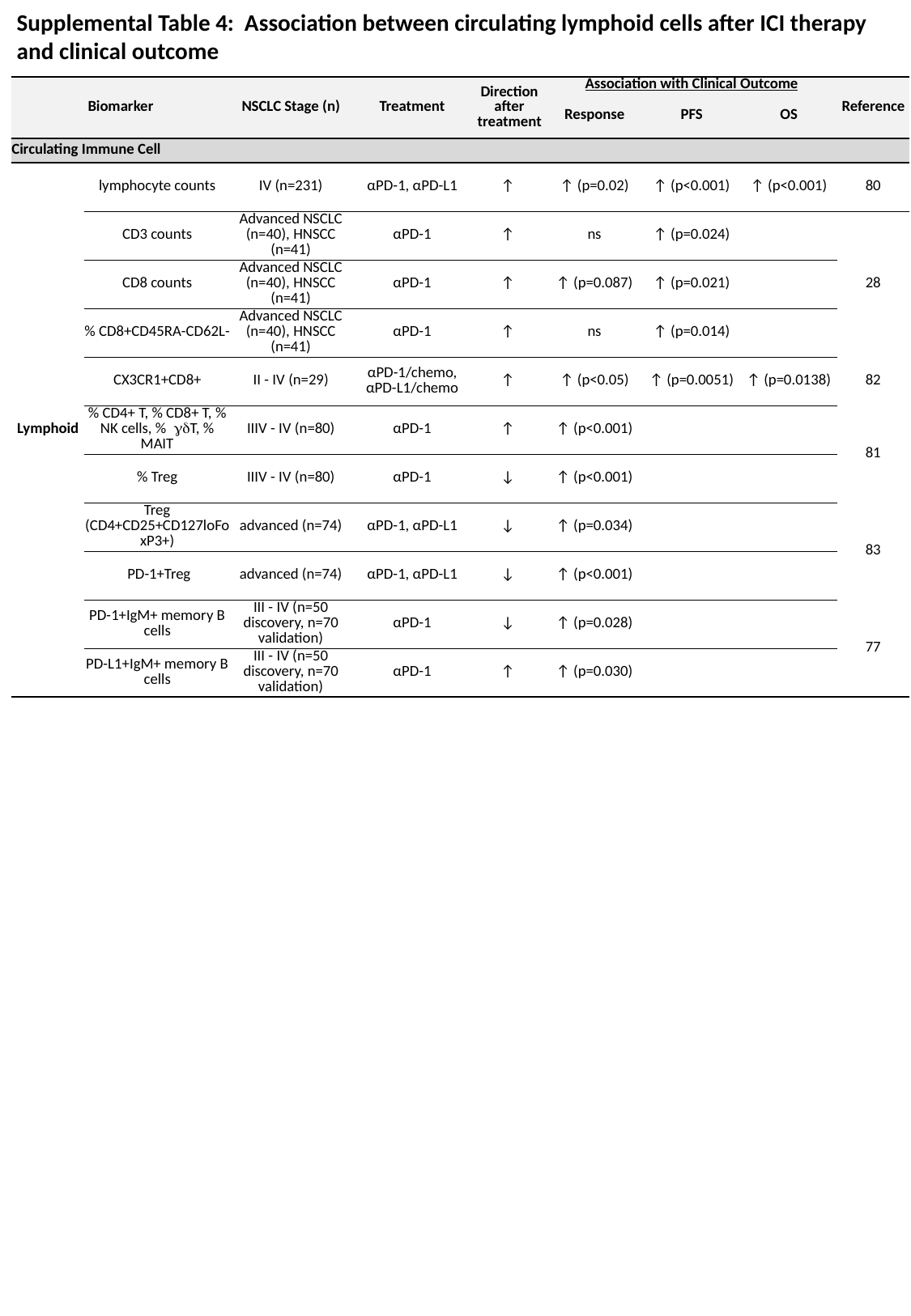

Supplemental Table 4: Association between circulating lymphoid cells after ICI therapy and clinical outcome
| Biomarker | | NSCLC Stage (n) | Treatment | Direction after treatment | Association with Clinical Outcome | | | Reference |
| --- | --- | --- | --- | --- | --- | --- | --- | --- |
| | | | | | Response | PFS | OS | |
| Circulating Immune Cell | | | | | | | | |
| Lymphoid | lymphocyte counts | IV (n=231) | αPD-1, αPD-L1 | ↑ | ↑ (p=0.02) | ↑ (p<0.001) | ↑ (p<0.001) | 80 |
| | CD3 counts | Advanced NSCLC (n=40), HNSCC (n=41) | αPD-1 | ↑ | ns | ↑ (p=0.024) | | 28 |
| | CD8 counts | Advanced NSCLC (n=40), HNSCC (n=41) | αPD-1 | ↑ | ↑ (p=0.087) | ↑ (p=0.021) | | |
| | % CD8+CD45RA-CD62L- | Advanced NSCLC (n=40), HNSCC (n=41) | αPD-1 | ↑ | ns | ↑ (p=0.014) | | |
| | CX3CR1+CD8+ | II - IV (n=29) | αPD-1/chemo, αPD-L1/chemo | ↑ | ↑ (p<0.05) | ↑ (p=0.0051) | ↑ (p=0.0138) | 82 |
| | % CD4+ T, % CD8+ T, % NK cells, % gdT, % MAIT | IIIV - IV (n=80) | αPD-1 | ↑ | ↑ (p<0.001) | | | 81 |
| | % Treg | IIIV - IV (n=80) | αPD-1 | ↓ | ↑ (p<0.001) | | | |
| | Treg (CD4+CD25+CD127loFoxP3+) | advanced (n=74) | αPD-1, αPD-L1 | ↓ | ↑ (p=0.034) | | | 83 |
| | PD-1+Treg | advanced (n=74) | αPD-1, αPD-L1 | ↓ | ↑ (p<0.001) | | | |
| | PD-1+IgM+ memory B cells | III - IV (n=50 discovery, n=70 validation) | αPD-1 | ↓ | ↑ (p=0.028) | | | 77 |
| | PD-L1+IgM+ memory B cells | III - IV (n=50 discovery, n=70 validation) | αPD-1 | ↑ | ↑ (p=0.030) | | | |

## Slide 5
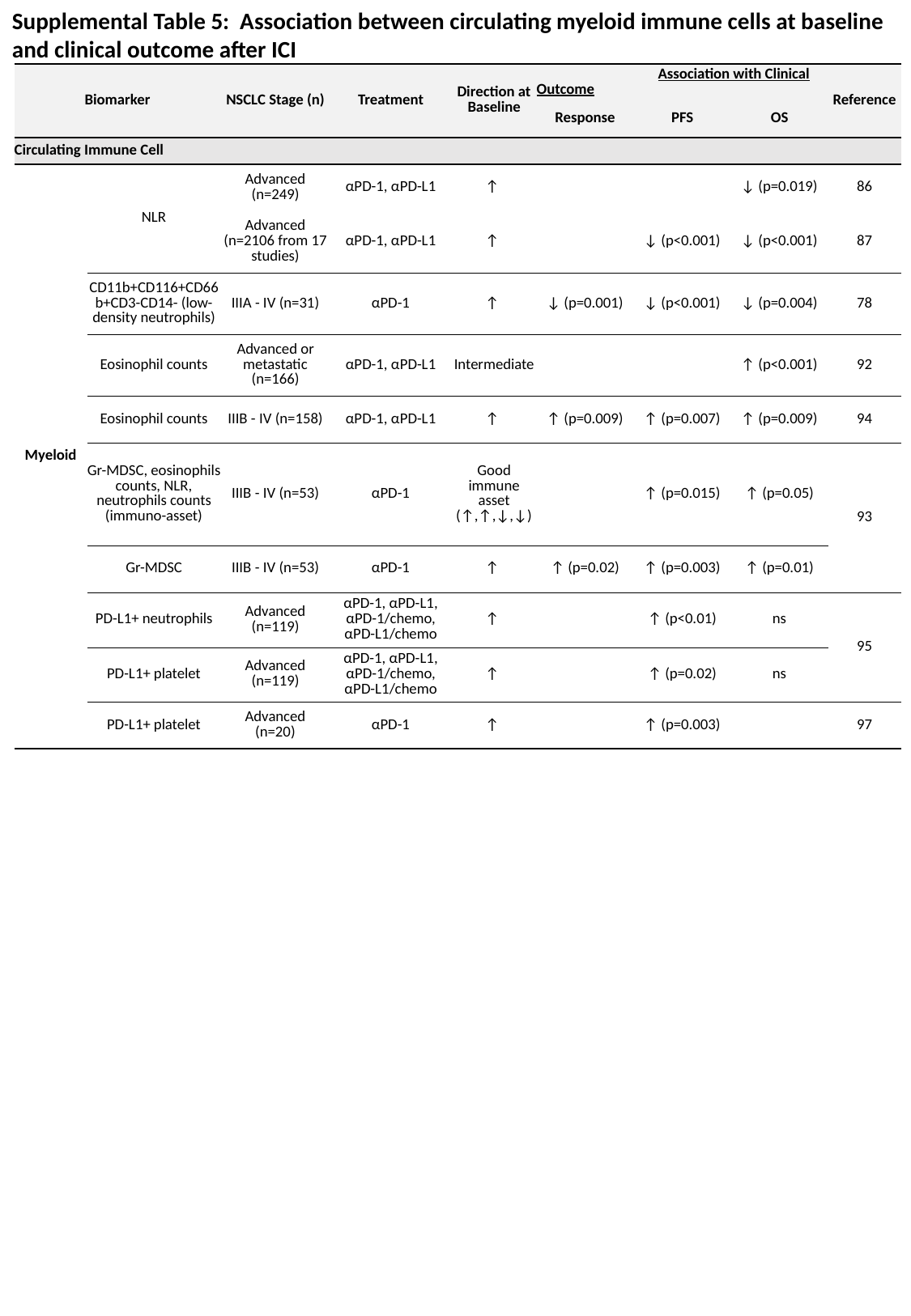

Supplemental Table 5: Association between circulating myeloid immune cells at baseline and clinical outcome after ICI
| Biomarker | | NSCLC Stage (n) | Treatment | Direction at Baseline | Association with Clinical Outcome | | | Reference |
| --- | --- | --- | --- | --- | --- | --- | --- | --- |
| | | | | | Response | PFS | OS | |
| Circulating Immune Cell | | | | | | | | |
| Myeloid | NLR | Advanced (n=249) | αPD-1, αPD-L1 | ↑ | | | ↓ (p=0.019) | 86 |
| | | Advanced (n=2106 from 17 studies) | αPD-1, αPD-L1 | ↑ | | ↓ (p<0.001) | ↓ (p<0.001) | 87 |
| | CD11b+CD116+CD66b+CD3-CD14- (low-density neutrophils) | IIIA - IV (n=31) | αPD-1 | ↑ | ↓ (p=0.001) | ↓ (p<0.001) | ↓ (p=0.004) | 78 |
| | Eosinophil counts | Advanced or metastatic (n=166) | αPD-1, αPD-L1 | Intermediate | | | ↑ (p<0.001) | 92 |
| | Eosinophil counts | IIIB - IV (n=158) | αPD-1, αPD-L1 | ↑ | ↑ (p=0.009) | ↑ (p=0.007) | ↑ (p=0.009) | 94 |
| | Gr-MDSC, eosinophils counts, NLR, neutrophils counts (immuno-asset) | IIIB - IV (n=53) | αPD-1 | Good immune asset (↑,↑,↓,↓) | | ↑ (p=0.015) | ↑ (p=0.05) | 93 |
| | Gr-MDSC | IIIB - IV (n=53) | αPD-1 | ↑ | ↑ (p=0.02) | ↑ (p=0.003) | ↑ (p=0.01) | |
| | PD-L1+ neutrophils | Advanced (n=119) | αPD-1, αPD-L1, αPD-1/chemo, αPD-L1/chemo | ↑ | | ↑ (p<0.01) | ns | 95 |
| | PD-L1+ platelet | Advanced (n=119) | αPD-1, αPD-L1, αPD-1/chemo, αPD-L1/chemo | ↑ | | ↑ (p=0.02) | ns | |
| | PD-L1+ platelet | Advanced (n=20) | αPD-1 | ↑ | | ↑ (p=0.003) | | 97 |

## Slide 6
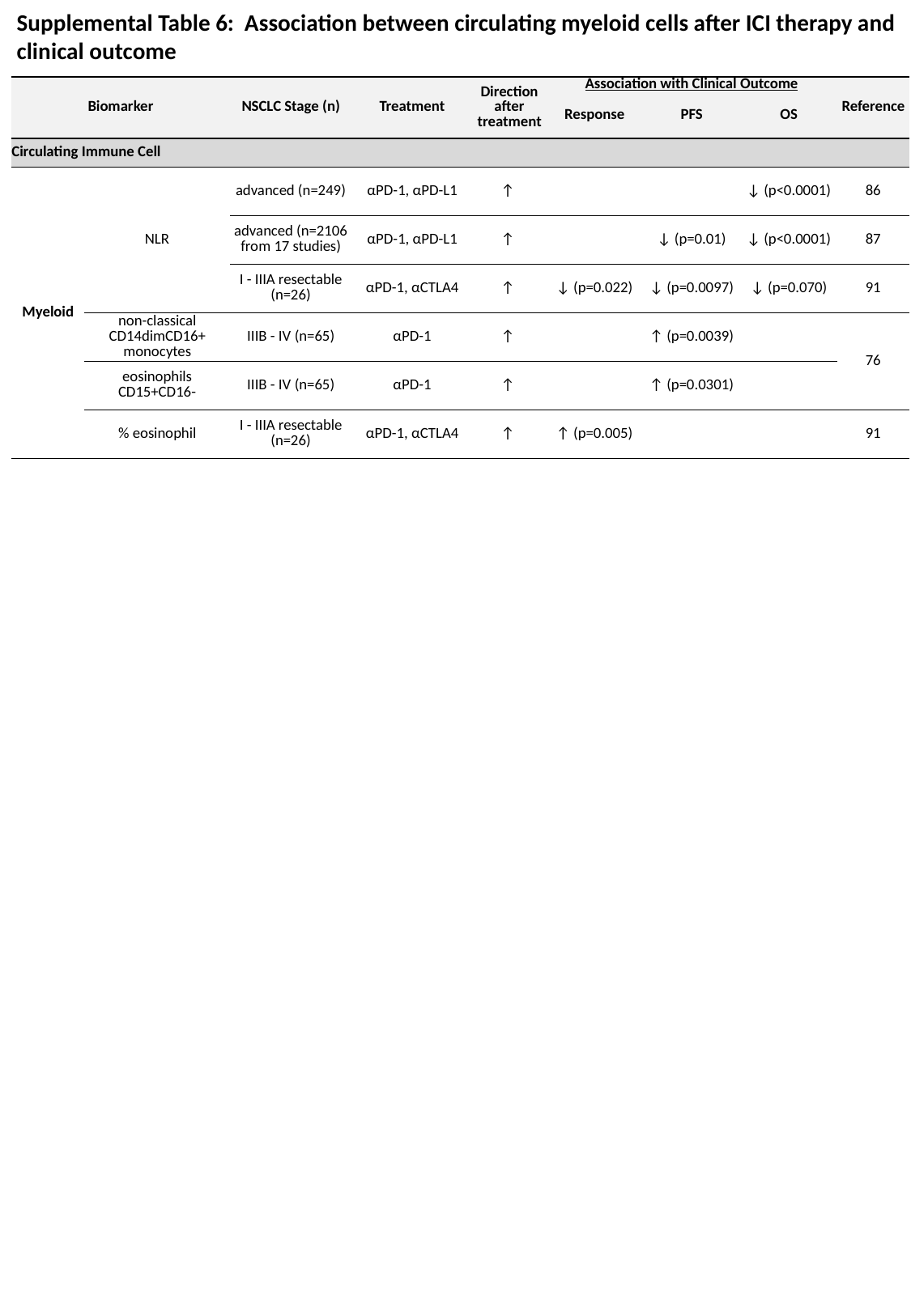

Supplemental Table 6: Association between circulating myeloid cells after ICI therapy and clinical outcome
| Biomarker | | NSCLC Stage (n) | Treatment | Direction after treatment | Association with Clinical Outcome | | | Reference |
| --- | --- | --- | --- | --- | --- | --- | --- | --- |
| | | | | | Response | PFS | OS | |
| Circulating Immune Cell | | | | | | | | |
| Myeloid | NLR | advanced (n=249) | αPD-1, αPD-L1 | ↑ | | | ↓ (p<0.0001) | 86 |
| | | advanced (n=2106 from 17 studies) | αPD-1, αPD-L1 | ↑ | | ↓ (p=0.01) | ↓ (p<0.0001) | 87 |
| | | I - IIIA resectable (n=26) | αPD-1, αCTLA4 | ↑ | ↓ (p=0.022) | ↓ (p=0.0097) | ↓ (p=0.070) | 91 |
| | non-classical CD14dimCD16+ monocytes | IIIB - IV (n=65) | αPD-1 | ↑ | | ↑ (p=0.0039) | | 76 |
| | eosinophils CD15+CD16- | IIIB - IV (n=65) | αPD-1 | ↑ | | ↑ (p=0.0301) | | |
| | % eosinophil | I - IIIA resectable (n=26) | αPD-1, αCTLA4 | ↑ | ↑ (p=0.005) | | | 91 |

## Slide 7
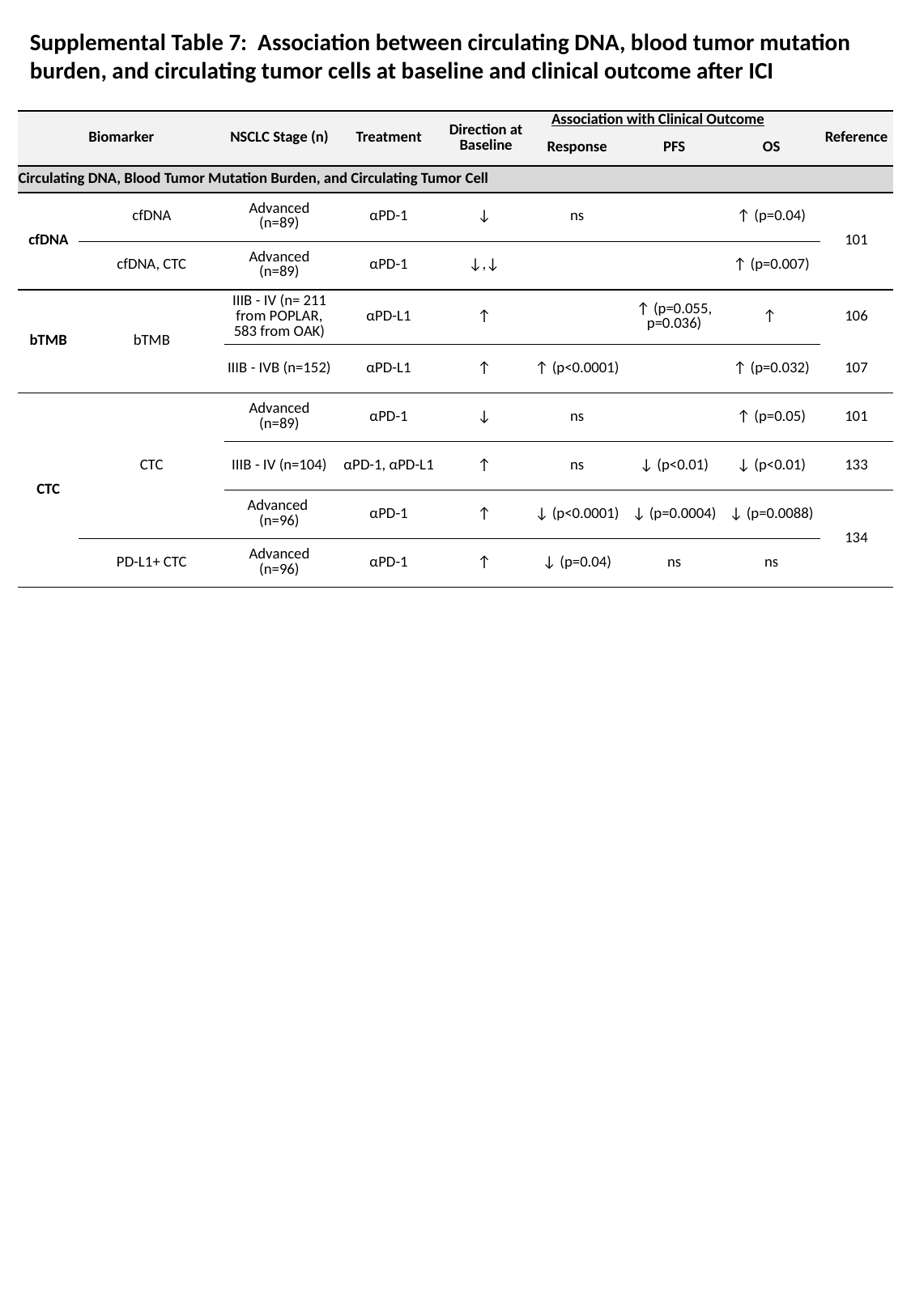

Supplemental Table 7: Association between circulating DNA, blood tumor mutation burden, and circulating tumor cells at baseline and clinical outcome after ICI
| Biomarker | | NSCLC Stage (n) | Treatment | Direction at Baseline | Association with Clinical Outcome | | | Reference |
| --- | --- | --- | --- | --- | --- | --- | --- | --- |
| | | | | | Response | PFS | OS | |
| Circulating DNA, Blood Tumor Mutation Burden, and Circulating Tumor Cell | | | | | | | | |
| cfDNA | cfDNA | Advanced (n=89) | αPD-1 | ↓ | ns | | ↑ (p=0.04) | 101 |
| | cfDNA, CTC | Advanced (n=89) | αPD-1 | ↓,↓ | | | ↑ (p=0.007) | |
| bTMB | bTMB | IIIB - IV (n= 211 from POPLAR, 583 from OAK) | αPD-L1 | ↑ | | ↑ (p=0.055, p=0.036) | ↑ | 106 |
| | | IIIB - IVB (n=152) | αPD-L1 | ↑ | ↑ (p<0.0001) | | ↑ (p=0.032) | 107 |
| CTC | CTC | Advanced (n=89) | αPD-1 | ↓ | ns | | ↑ (p=0.05) | 101 |
| | | IIIB - IV (n=104) | αPD-1, αPD-L1 | ↑ | ns | ↓ (p<0.01) | ↓ (p<0.01) | 133 |
| | | Advanced (n=96) | αPD-1 | ↑ | ↓ (p<0.0001) | ↓ (p=0.0004) | ↓ (p=0.0088) | 134 |
| | PD-L1+ CTC | Advanced (n=96) | αPD-1 | ↑ | ↓ (p=0.04) | ns | ns | |

## Slide 8
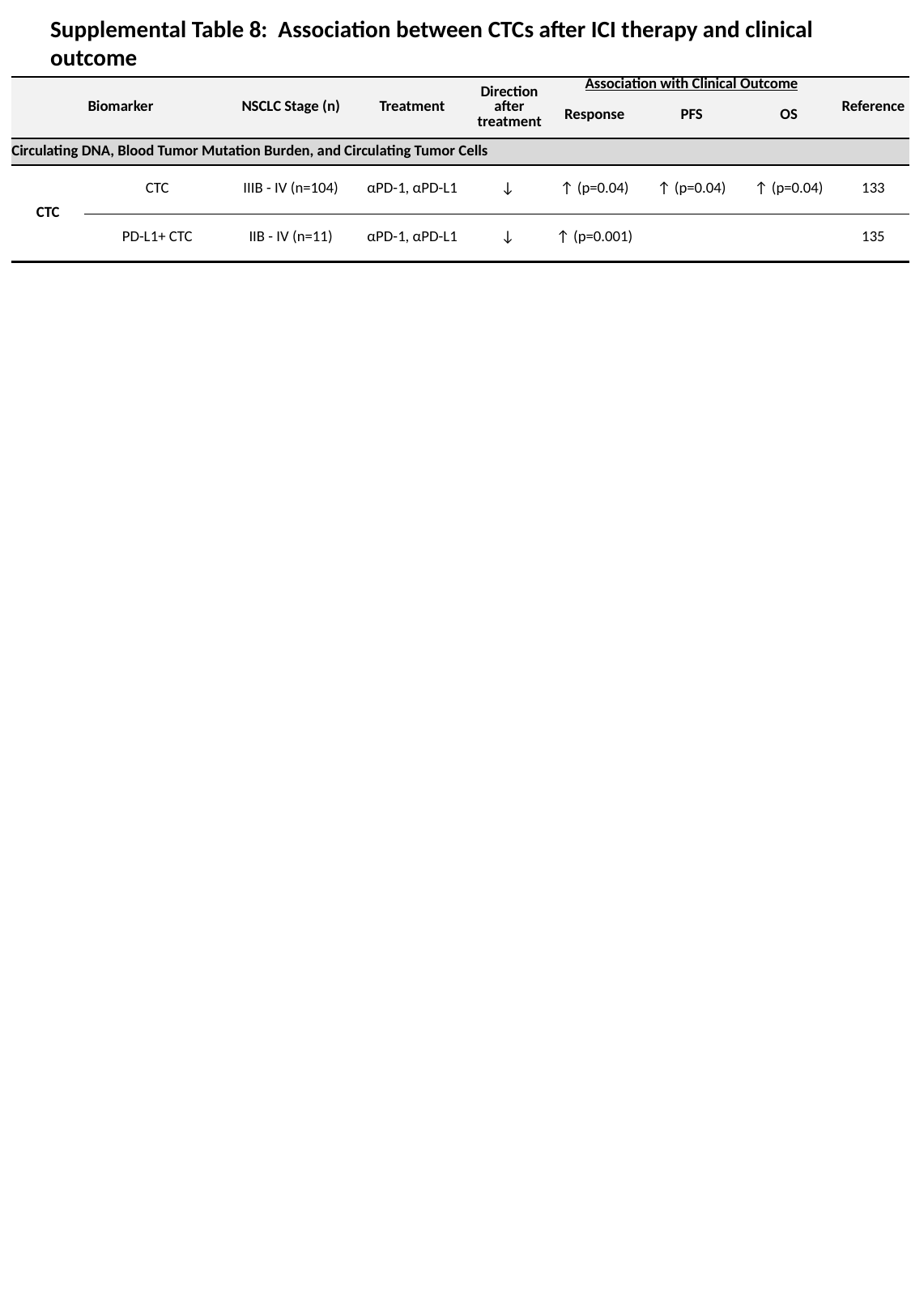

Supplemental Table 8: Association between CTCs after ICI therapy and clinical outcome
| Biomarker | | NSCLC Stage (n) | Treatment | Direction after treatment | Association with Clinical Outcome | | | Reference |
| --- | --- | --- | --- | --- | --- | --- | --- | --- |
| | | | | | Response | PFS | OS | |
| Circulating DNA, Blood Tumor Mutation Burden, and Circulating Tumor Cells | | | | | | | | |
| CTC | CTC | IIIB - IV (n=104) | αPD-1, αPD-L1 | ↓ | ↑ (p=0.04) | ↑ (p=0.04) | ↑ (p=0.04) | 133 |
| | PD-L1+ CTC | IIB - IV (n=11) | αPD-1, αPD-L1 | ↓ | ↑ (p=0.001) | | | 135 |
